# High variability exists in 3D leg alignment analysis, but underlying principles that might lead to agreement on a universal framework could be identified: A systematic review

**DOI:** 10.1002/ksa.12512

**Published:** 2024-10-26

**Authors:** Quinten W. T. Veerman, Romy M. ten Heggeler, Gabriëlle J. M. Tuijthof, Feike de Graaff, René Fluit, Roy A. G. Hoogeslag

**Affiliations:** ^1^ OCON Centre for Orthopaedic Surgery and Sports Medicine Hengelo the Netherlands; ^2^ Faculty of Engineering Technology University of Twente Enschede the Netherlands; ^3^ Faculty of Science and Engineering University of Groningen Groningen the Netherlands

**Keywords:** alignment parameters, coordinate systems, knee, osteotomy, TKA

## Abstract

**Purpose:**

To (1) investigate the hypothesis that there is high variability in the reported methods to derive axes and joint orientations from three‐dimensional (3D) bone models to (a) perform 3D knee‐related leg alignment analysis and (b) define coordinate systems for the femur, tibia and leg and (2) identify underlying principles that might lead to agreement on a universal 3D leg alignment analysis framework.

**Methods:**

A systematic review of the literature between January 2006 and June 2024 was performed. Articles explicitly reporting methods to derive axes and joint orientations from CT‐based 3D bone models for alignment parameters and/or coordinate systems of the femur, tibia and leg were included. Study characteristics and reported methods were extracted and presented as a qualitative synthesis.

**Results:**

A total of 93 studies were included. There was high variability in the reported methods to derive axes and joint orientations from 3D bone models. Nevertheless, the reported methods could be categorized into four groups, and several underlying principles of the four groups could be identified. Furthermore, the definitions of femoral and tibial coordinate systems were most frequently based on the mechanical axis (femoral, 13/19 [68%]; tibial, 13/26 [50%]) and a central medial‐lateral axis (femoral, 16/19 [84%]; tibial, 12/26 [46%]); no leg coordinate system was reported. Interestingly, of the included studies that reported on leg alignment parameters (76/93, 82%), only a minority reported expressing these in a complete coordinate system (25/76, 33%).

**Conclusion:**

There is high variability in 3D knee‐related leg alignment analysis. Therefore, universal 3D reference values for alignment parameters cannot yet be defined, and comparison of alignment parameter values between different studies is impossible. However, several underlying principles to the reported methods were identified, which could serve to reach more agreement on a future universal 3D framework for leg alignment analysis.

**Level of Evidence:**

Level I.

**Abbreviations**
2D: two‐dimensional
3D: three‐dimensional
CA: critical appraisal
CT: computed tomography
FCA: femoral condylar axis
ICC: intraclass correlation coefficient
ISB: International Society of Biomechanics
LOE: level of evidence
mFA: mechanical femoral axis
MRI: magnetic resonance imaging
mTA: mechanical tibial axis
PRISMA: Preferred Reporting Items for Systematic Reviews and Meta Analyses
PTJ ML: proximal medial‐lateral tibial joint orientation

## INTRODUCTION

In recent years, both for osteotomies around the knee and total knee arthroplasty, there is an increasing interest in leg alignment analysis not only in the coronal plane but also in the sagittal and axial planes [27, 46, 89, 92, 103].

For the current gold standard two‐dimensional (2D) framework for leg alignment analysis, a high degree of consensus exists on how to derive axes and joint orientations that are necessary to define joint alignment and joint orientation angles from the radiographs and torsion/version angles from the 2D computed tomography (CT) slices [65, 81]. However, the 2D framework simplifies the complex 3D shapes of bones and articular surfaces; it introduces errors due to inadequate leg and knee positioning and image distortion [38, 73, 76]; and moreover, it prevents accurate analysis of three‐dimensional (3D) multiplanar deformities in their separate (not necessarily orthogonal) coronal, sagittal and axial 2D radiographic projections [93].

By contrast, through the generation of 3D bone models, 3D imaging modalities allow for a more accurate assessment of leg malalignment without further simplifications of reality [31]. It was even proposed that ‘the time has come’ [17] to transfer completely from a 2D to a 3D framework for malalignment analysis and correction planning of osteotomies around the knee. This indicates the need for a universal 3D framework on how to accurately and reliably derive axes and joint orientations from 3D bone models. However, since this is an emerging field of interest, and (in contrast to the 2D framework) the surface data on a 3D bone model from which to derive axes and joint orientations is abundant, it is hypothesized that consensus on such a 3D framework for leg alignment analysis is absent [22]. This lack of consensus could hinder the establishment of 3D reference values for alignment parameters, which, from a clinical point of view, would render the comparability between alignment parameter values of different studies impossible, and might lead to an inappropriate correction in osteotomy around the knee [22] or positioning of a total knee prosthesis.

Therefore, the purpose of this systematic review was to critically appraise, summarize, and compare literature on 3D frameworks for knee‐related leg alignment analysis. Our aims were to (1) investigate the hypotheses that there is a high variability in the reported methods to derive axes and joint orientations from 3D bone models to (a) perform 3D knee‐related leg alignment analysis and (b) define coordinate systems for the femur, tibia, and leg and (2) identify underlying principles that might lead to agreement on a universal 3D leg alignment analysis framework.

## METHODS

The PRISMA (Preferred Reporting Items for Systematic Reviews and Meta‐Analyses) 2020 guidelines were followed, and a PRISMA 2020 flow diagram was used (PROSPERO ID:[CRD42022303250]).

### Search strategy

On 7 June 2024, one author (QV) performed a comprehensive literature search in the Scopus, MEDLINE/PubMed and Embase databases. Search strings were used to screen for studies that reported on methods to derive axes and/or joint orientations from 3D bone models for alignment parameters and/or coordinate systems of the femur, tibia, and leg (Supporting Information S1: Table 1). Considering the technical progress of 3D imaging techniques, the search was limited to studies published since 1 January 2006.

### Inclusion and exclusion criteria

**Figure 1:**
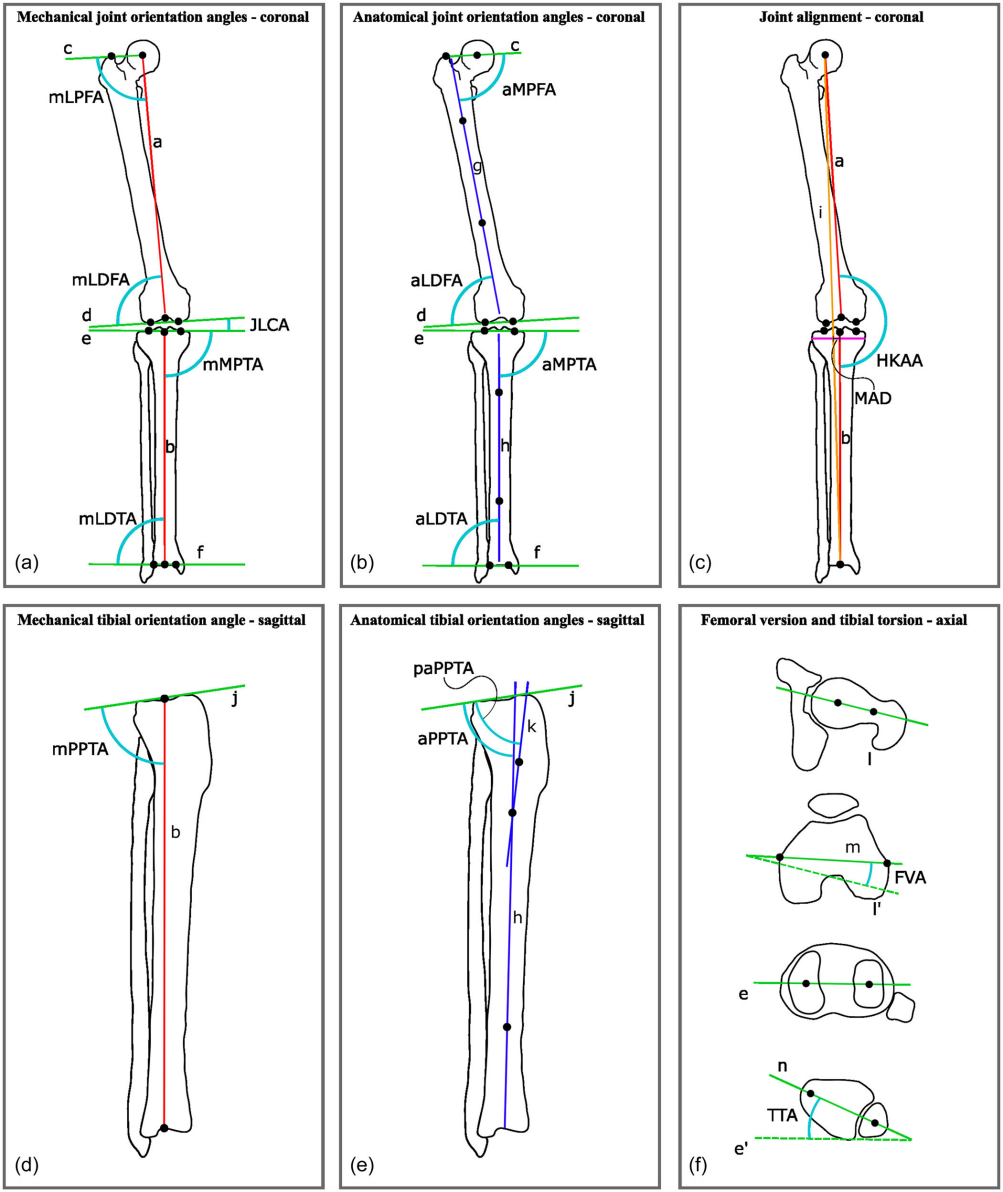
Schematic representation of axes and joint orientations from relevant knee‐related alignment parameters [82] on one of three anatomical reference planes. (a) Projections of joint orientations and mechanical axes of the femur and tibia on the coronal plane, and resulting mechanical joint orientation angles in the coronal plane. (b) Projections of joint orientation and anatomical axes of the femur and tibia on the coronal plane, and anatomical joint orientation angles in the coronal plane. (c) Projections of mechanical axes of the femur, tibia and leg on the coronal plane, and leg joint alignment in the coronal plane. (d) Projection of joint orientation and mechanical axes of the tibia on the sagittal plane, and mechanical joint orientation angle in the sagittal plane. (e) Projection of joint orientation and anatomical axes of the tibia on the sagittal plane, and anatomical joint orientation angles of the tibia in the sagittal plane [14]. (f) Projection of joint orientation on the femoral and tibial axial plane, as well as femoral version and tibial torsion angles in the femoral and tibial axial plane. Joint orientation and axes: (a, mechanical femoral axis; b, mechanical tibial axis; c, hip joint orientation; d, distal femoral joint orientation; e, proximal medial‐lateral tibial joint orientation; f, distal medial‐lateral tibial joint orientation; g, anatomical femoral axis; h, anatomical tibial axis; i, mechanical leg axis; j, proximal anterior‐posterior tibial joint orientation; k, anatomical proximal tibial axis; l, femoral neck axis; m, femoral (epi)condylar axis; n, tibial intermalleolar axis. aLDFA, anatomical lateral distal femoral angle; aLDTA, anatomical lateral distal tibial angle; aMPFA, anatomical medial proximal femoral angle; aMPTA, anatomical medial proximal tibial angle; aPPTA, anatomical posterior proximal tibial angle; FVA, femoral version angle; HKAA, hip‐knee‐ankle‐angle; JLCA, joint‐line convergence angle; MAD, mechanical axis deviation; mLDFA, mechanical lateral distal femoral angle; mLDTA, mechanical lateral distal tibial angle; mLPFA, mechanical lateral proximal femoral angle; mMPTA, mechanical medial proximal tibial angle; mPPTA; mechanical posterior proximal tibial angle; paPPTA, proximal anatomical posterior proximal tibial angle; TTA, tibial torsion angle.

The inclusion and exclusion criteria are reported in Table 1. Only papers that used CT‐based 3D models were included since this is the preferred imaging modality for segmentation purposes due to its relative homogeneous bone signal intensities [53]. Furthermore, the axes and joint orientations of alignment parameters that were deemed relevant for the planning of an osteotomy around the knee [14, 82] are reported in Figure 1.

**Table 1:** Inclusion and exclusion criteria applied to eligible articles.

Inclusion	1) Human subjects with closed epiphysis (≥18 years of age); 2) Original and peer‐reviewed; 3) Focused on the knee joint; 4) Investigated native and intact bone; 5) Reported their analysis on CT‐based 3D bone models; 6) Reported a method to derive one or more axes or joint orientations for relevant knee‐related alignment parameters, and/or 7) Reported a method to derive one or more axes that define a Cartesian coordinate system of the femur, tibia and/or leg.
Exclusion	1) Languages other than English, Dutch, or German

Abbreviations: 3D, three‐dimensional; CT, computed tomography.

### Review process

After removal of duplicates, two reviewers (QV and RtH) independently screened titles and abstracts of the identified studies for potential eligibility [80] and evaluated the full texts of studies eligible for inclusion. Disagreement on in‐ or exclusion was resolved by a third reviewer (RH). The references of included studies were cross‐checked for in‐ and exclusion criteria (Table 1) independently by two authors (QV and RtH) to find studies that were not identified in the original search.

### Data extraction

Two reviewers (QV and RtH) independently extracted, summarized and tabulated the study characteristics and the reported methods to derive axes or joint orientations from 3D bone models for knee‐related alignment parameters and/or coordinate systems for the femur, tibia and leg and the reliability of the derived alignment parameters (if reported).

Coordinate systems were categorized as ‘complete’ if implicitly or explicitly a coordinate system with three orthogonal axes and an origin could be derived. Extracted data were tabulated in Excel version nr. 2203 (Microsoft Corporation).

### Quality assessment

Two reviewers (QV and RtH) independently rated the level of evidence (LOE) and performed a critical appraisal (CA). The LOE and study design were rated according to the Oxford Centre of Evidence‐Based Medicine [34]. The CA was performed with the Quality Assessment Tool for Observational Cohort and Cross‐Sectional Studies from the National Institutes of Health [13, 77]. This tool [77] consists of fourteen questions to assess the internal validity and methodological quality of each study, including potential bias and confounders [13]. With this tool, exposure of interest was defined as leg alignment assessed in 2D, and outcomes were defined as leg alignment assessed on 3D bone models.

### Statistical analysis

The results of the reported methods to derive axes or joint orientations from 3D bone models are presented as a qualitative synthesis. First, the reported methods were categorized per segment (tibia, femur or leg). Then, per segment, the methods were categorized under one of the axes or joint orientations of interest (Figure 1). Finally, to obtain a higher level of abstraction, all reported methods were investigated to find a common approach or principle and were grouped accordingly. The results of the intraclass correlation coefficients (ICCs) of the reported methods within the included studies are presented with their 95% confidence intervals. Categorical data were reported as frequencies and percentages.

## RESULTS

### Search results

**Figure 2:**
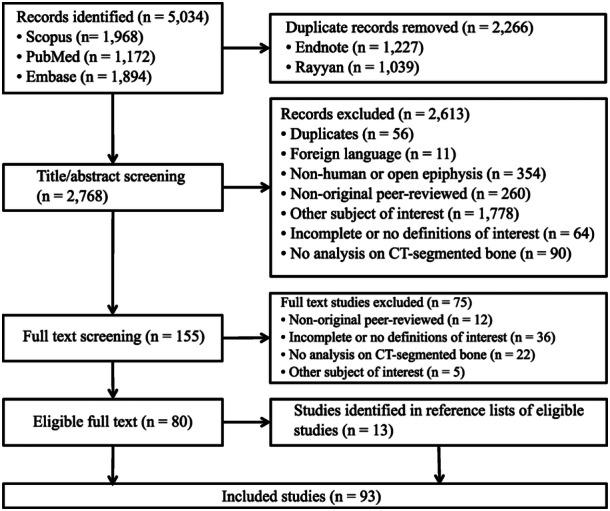
PRISMA 2020 flow chart for screening and inclusion of studies. Studies could have been excluded on the basis of multiple criteria, with the first reason for exclusion reported. PRISMA, Preferred Reporting Items for Systematic Reviews and Meta‐Analyses.

The search of the Scopus, MEDLINE/PubMed and Embase databases provided 5034 citations, of which 2266 duplicates were removed. After exclusion, a total of 93 studies were included in this review (Figure 2).

### Study characteristics, LOE and critical appraisal

The study characteristics are presented in Supporting Information S1: Table 2. The majority of included studies were Oxford LOE III (*n* = 75 studies) [3, 4, 5, 6, 7, 8, 9, 10, 11, 15, 16, 18, 19, 20, 21, 23, 25, 28, 29, 30, 31, 32, 33, 35, 36, 37, 39, 41, 44, 47, 48, 49, 50, 51, 52, 54, 55, 56, 57, 58, 59, 60, 61, 62, 63, 64, 66, 68, 69, 70, 78, 79, 83, 84, 85, 87, 88, 90, 91, 94, 96, 97, 98, 99, 100, 101, 102, 105, 110, 111, 112, 113, 114, 115, 116], and 18 studies were rated Oxford LOE IV [1, 2, 12, 22, 24, 42, 43, 45, 71, 72, 74, 75, 86, 95, 104, 106, 107, 109] (Supporting Information S1: Table 2). Of the included studies, 16 were human cadaver studies [2, 5, 7, 10, 12, 15, 16, 20, 22, 24, 30, 39, 71, 75, 104, 106], 48 were retrospective cohort studies [3, 4, 6, 8, 9, 11, 19, 21, 23, 25, 28, 29, 31, 32, 33, 35, 37, 41, 43, 44, 48, 49, 50, 51, 52, 59, 60, 61, 62, 63, 64, 68, 69, 70, 83, 85, 86, 87, 88, 94, 96, 97, 98, 99, 102, 107, 112, 115], 23 were prospective cohort studies [18, 36, 42, 45, 47, 54, 55, 56, 57, 58, 74, 79, 84, 90, 91, 100, 101, 105, 110, 111, 113, 114, 116], 2 were case‐control studies [72, 78], 2 were case studies [95, 109], 1 investigated a mixed retrospective cohort of human cadavers and subjects [66] and 1 used a knee joint model [1] (Supporting Information S1: Table 3). The critical appraisal is presented in Supporting Information S1: Table 3.

### Outcomes

#### Reported methods on how to derive axes and joint orientations from 3D bone models

**Figure 3:**
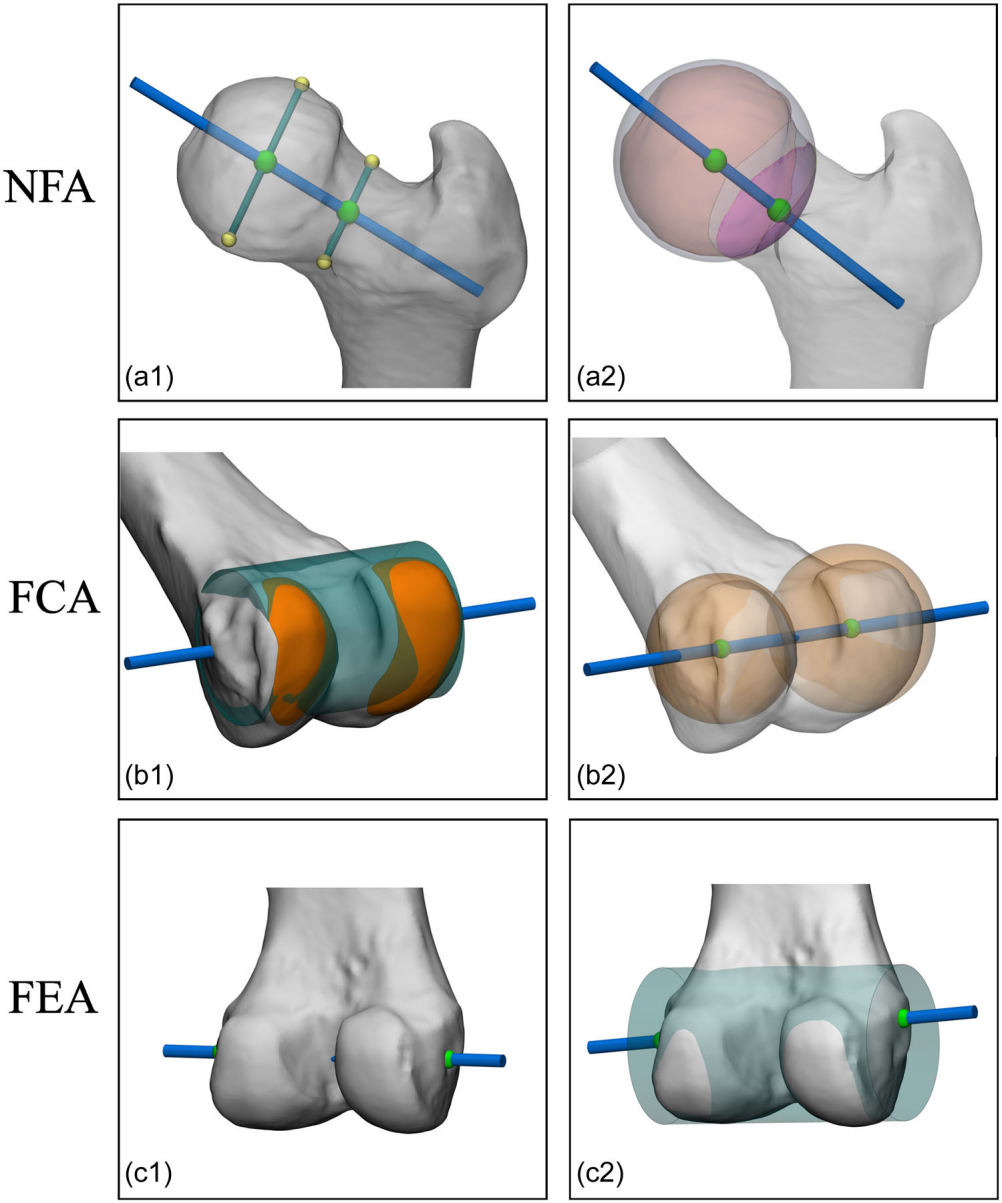
Examples of the variability of reported methods to derive femoral axes and joint orientations from 3D bone models (used for knee‐related femoral alignment parameters and coordinate systems). (a1) Axis connecting midpoints of manually selected anterior and posterior points of (1) femoral head and (2) femoral neck [22]. (a2) Axis connecting the centre of the sphere fitted to the femoral head with the centre of the intersection at the base of the femoral neck, using a 2 mm radius‐increased sphere fitted to the femoral head [115]. (b1) Axis of the cylinder fitted to the marked flexion‐extension surfaces of the distal femur [84]. (b2) Axis connecting midpoints of sphere‐fit medial and lateral femoral condyles [9]. (c1) Axis connecting the manually selected most concave point medial femoral epicondyle with the most prominent point lateral femoral epicondyle [52, 64, 69, 99, 106, 112]. (c2) Axis connecting the spot of the bone visible on medial and lateral condyles after extension of the cylinder used in B3 [16]. FCA, femoral condylar axis; FEA, femoral epicondylar axis; NFA, neck femur axis.

**Figure 4:**
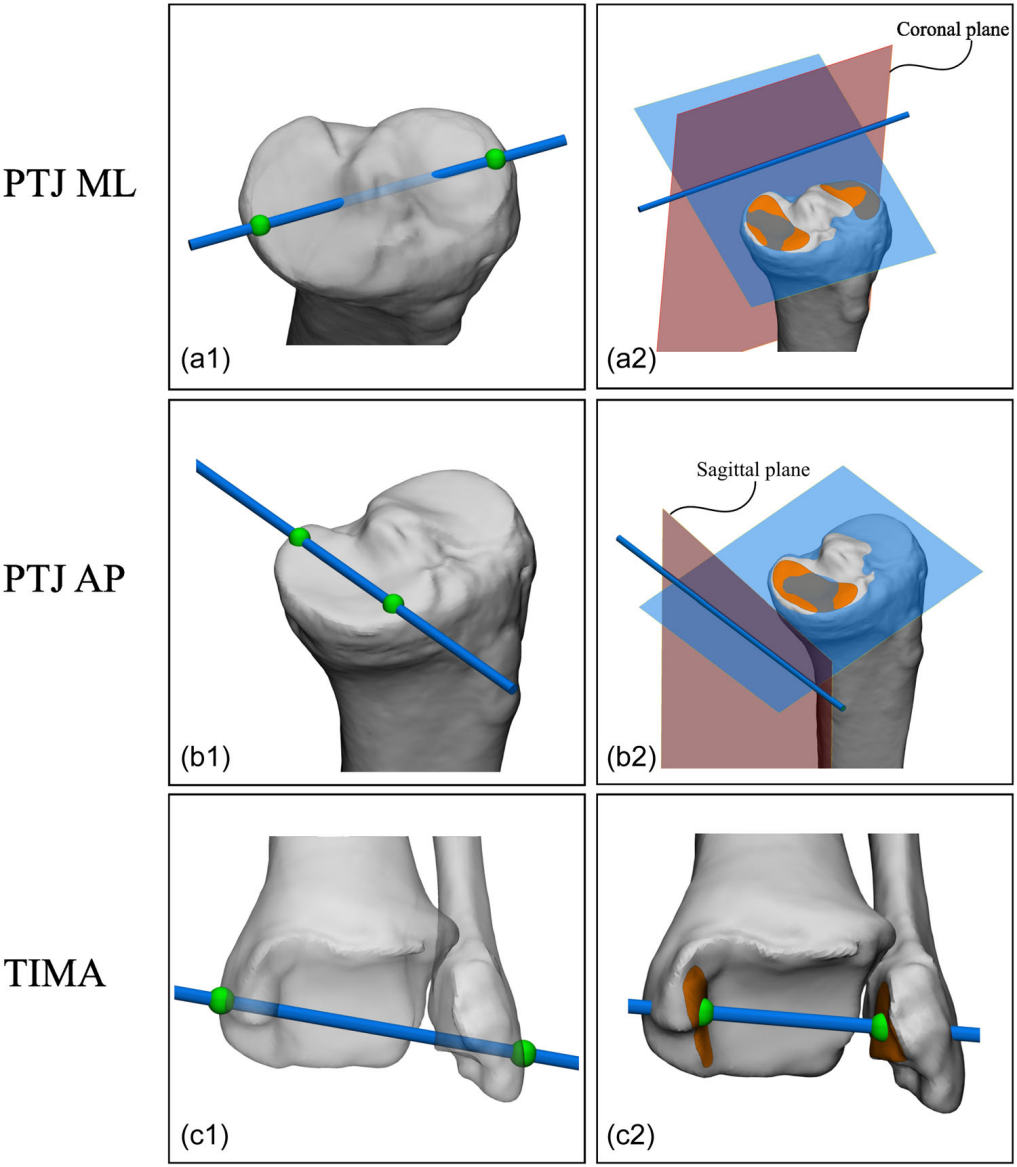
Examples of the variability of reported methods to derive tibial axes and joint orientations from 3D bone models (used for knee‐related tibial alignment parameters and coordinate systems). (a1) Axis connecting manually selected single most medial and most lateral tibial plateau points [6, 22, 95, 99]. (a2) Best‐fit plane through the marked articular surfaces of the medial and lateral tibial plateau indicates proximal tibial plateau 3D joint orientation; the intersecting line between this plane and the tibial coronal plane indicates the medial‐lateral orientation of the tibial plateau joint surface [28]. (b1) Axis connecting the manually selected single most cranial and anterior point with the most cranial and posterior point of the medial tibia plateau [22, 39, 68]. (b2) Best‐fit plane through the marked articular surface of the medial tibial plateau indicates proximal medial tibial plateau 3D joint orientation; the intersecting line between this plane and the tibial sagittal plane indicates the anterior–posterior orientation of the medial tibial plateau joint surface [29, 96]. (c1) Axis connecting manually selected most medial and lateral points of medial and lateral malleoli, respectively [22]. (c2) Axis connecting midpoints of articular surfaces of the medial and lateral malleoli [42]. PTJ AP, proximal tibial joint orientation anterior‐posterior; PTJ ML, proximal tibial joint orientation medial‐lateral; TIMA, tibial intermalleolar axis.

Overall, there was high variability in the reported methods on how to derive axes and joint orientations from 3D bone models for knee‐related leg alignment parameters and femoral and tibial/fibular coordinate systems (Supporting Information S1: Tables 4–6, respectively). This ranged from the use of a line through two arbitrary points on the 3D bone model's surface to the use of all available relevant surface data on a 3D bone model (Figures 3 and 4).

**Figure 5:**
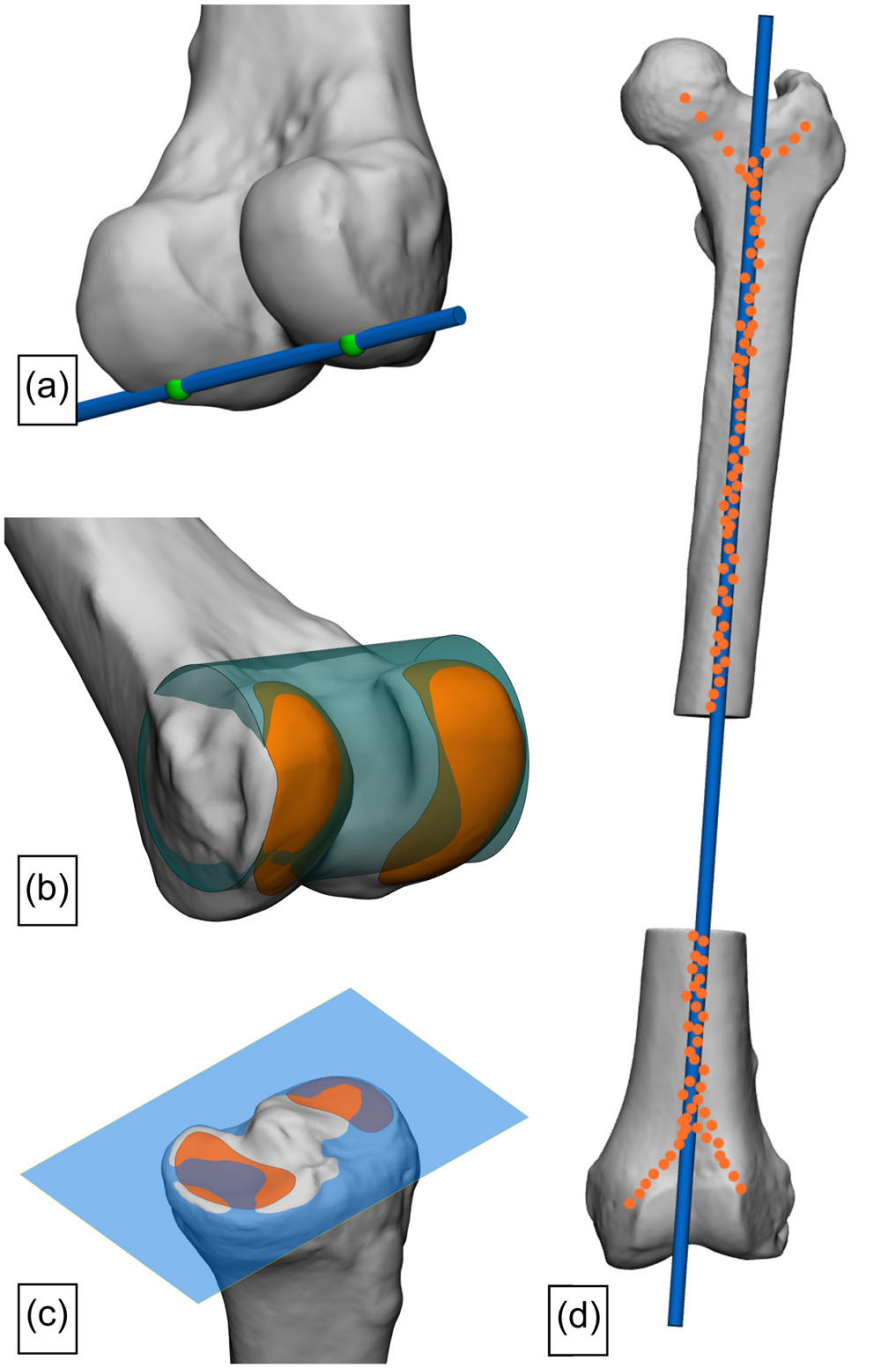
Four groups in which the reported methods to derive axes or joint orientations from 3D bone models could be categorized, with examples per group. (a) Line between two landmark points (Landmark method; for example, distal femoral joint orientation). (b) Geometrical shape (e.g., cylinder) with the best fit to a multitude of points on the round articular surface (best‐fit geometrical shape method, e.g., femoral condylar axis) from which a central axis can be extracted. (c) A plane fitting a multitude of points on a flat articular surface (blue; calculated plane method; e.g., proximal tibial joint orientation) from which either the normal or the intersecting line with an anatomical reference plane can be extracted. (d) Line calculated from a multitude of points (calculated line method, e.g., anatomical femoral axis).

Nevertheless, the reported methods could be categorized into four groups: (1) a ‘Landmark method’ [3, 4, 5, 6, 7, 8, 9, 10, 12, 15, 16, 18, 19, 20, 21, 22, 23, 24, 25, 28, 29, 30, 32, 33, 35, 37, 39, 41, 42, 43, 44, 47, 49, 51, 52, 54, 55, 56, 57, 58, 59, 60, 61, 62, 63, 64, 66, 68, 69, 70, 78, 79, 83, 87, 88, 91, 94, 95, 96, 97, 98, 99, 100, 101, 102, 104, 106, 107, 109, 110, 112, 113, 114, 115, 116], (2) a ‘Calculated line method’ [5, 10, 19, 20, 31, 35, 41, 44, 66, 74, 87, 95, 97, 109, 113], (3) a ‘Calculated plane method’ [2, 4, 18, 28, 29, 31, 33, 37, 41, 44, 70, 83, 87, 88, 94, 96] or (4) a ‘Best‐fit geometrical shape method’ [8, 16, 20, 24, 47, 84] (Figure 5).

Moreover, although the Landmark method was used to derive all 3D axes and joint orientations reported in the included studies [5, 6, 8, 9, 10, 12, 15, 16, 18, 20, 21, 22, 23, 24, 25, 28, 29, 30, 37, 39, 41, 42, 43, 47, 49, 51, 52, 54, 55, 56, 57, 58, 61, 63, 64, 66, 68, 69, 70, 78, 79, 83, 87, 91, 94, 95, 96, 98, 99, 100, 101, 102, 104, 106, 109, 110, 112, 113, 114, 115, 116], for the other groups it could be distinguished that (1) the calculated line method was more specifically used to derive anatomical femoral and tibial axes [5, 10, 18, 20, 31, 41, 66, 74, 87, 95, 109], (2) the calculated plane method was more specifically used to derive all joint orientations that originate from the proximal tibial joint surfaces [2, 28, 29, 31, 37, 41, 70, 83, 87, 94, 96], and (3) the best‐fit geometrical shape method was more specifically used to derive the femoral condylar axis [16, 24, 84] and neck femur axis [47].

#### Coordinate systems

Complete femoral and/or tibial coordinate systems (Supporting Information S1: Tables 7 and 8, respectively) were reported in 38/93 (41%) [1, 2, 3, 6, 8, 11, 18, 20, 35, 36, 42, 44, 45, 48, 50, 52, 57, 60, 62, 63, 64, 66, 71, 72, 74, 75, 79, 86, 90, 91, 98, 104, 105, 110, 111, 113] of the included studies (*n* = 19 femoral and *n* = 26 tibial); for the leg, no complete coordinate system was reported.

The definition of both the femoral and tibial coordinate systems was most frequently based on their respective mechanical axes (femoral, 13/19 [68%] [1, 6, 45, 52, 57, 60, 63, 64, 72, 79, 86, 91, 104], tibial, 13/26 (50%) [3, 8, 18, 44, 62, 66, 72, 86, 90, 98, 104, 110, 111]) and central medial‐lateral axis (femoral, 16/19 [84%] [1, 6, 20, 35, 36, 45, 52, 57, 60, 63, 64, 71, 72, 79, 86, 104] and tibial, 12/26 [46%] [2, 3, 8, 11, 36, 44, 45, 71, 86, 104, 111, 113]). Additionally, for the tibial coordinate system, a relevant minority of the reported studies based the longitudinal axis on the normal to a best‐fit plane of (the articular surface of) the tibial plateau (4/26 [15%]) [11, 48, 50, 74].

Interestingly, while 76/93 (82%) of the included studies [2, 5, 6, 7, 8, 9, 10, 12, 15, 16, 18, 19, 20, 21, 22, 23, 24, 25, 28, 29, 30, 31, 32, 33, 37, 40, 41, 42, 43, 44, 47, 48, 49, 50, 51, 52, 54, 55, 56, 57, 59, 60, 61, 62, 63, 64, 66, 68, 69, 70, 74, 78, 79, 83, 87, 88, 90, 91, 94, 95, 96, 97, 98, 100, 101, 102, 104, 106, 109, 110, 111, 112, 113, 114, 115, 116] reported alignment parameters, only 25 of these 76 studies (33%) [2, 6, 8, 18, 20, 42, 44, 48, 50, 52, 57, 60, 62, 63, 64, 66, 74, 79, 90, 91, 98, 104, 110, 111, 113] reported to express these in (an anatomical reference plane of) a complete coordinate system.

#### Reliability of the reported methods to derive alignment parameters from 3D bone models

The ICC for alignment parameters was reported in 22/93 (24%) of the included studies [2, 8, 22, 24, 28, 29, 31, 41, 42, 43, 49, 54, 59, 60, 63, 74, 87, 90, 98, 110, 114, 116], and all but three studies [22, 24, 110] reported good to excellent reliability for both manual and (semi‐)automatic methods to derive alignment parameters from 3D bone models (Supporting Information S1: Table 9).

## DISCUSSION

The most important findings of this study are (1) that overall, there is high variability in reported methods on how to derive axes and joint orientations from CT‐based 3D bone models to (a) perform 3D knee‐related leg alignment analysis and (b) define coordinate systems with their resulting anatomical reference planes in which alignment parameters are expressed, for the femur and tibia; (2) that only a minority (33%) of the studies that reported on leg alignment also reported complete coordinate systems with their corresponding anatomical reference plane in which alignment parameters are to be expressed; and (3) that despite these findings several underlying principles to the reported methods could be identified. These findings are largely in line with our hypothesis.

In order to transition from 2D to 3D leg alignment analysis and correction planning, one would expect agreement on a universal framework on how to derive axes and joint orientations from a 3D bone model. But as the results of the present study clearly show, this is not the case. One implication of this is that it renders the comparability between studies with a 3D framework for leg alignment analysis nearly impossible. The variability in reported methods to derive axes and joint orientations from 3D bone models for leg alignment parameters could lead to different values per alignment parameter between studies, even if projected on identical coordinate systems. Additionally, the variability in methods to derive axes from 3D bone models for complete coordinate systems could lead to different alignment‐parameter values when calculating the same alignment parameters on anatomical reference planes of coordinate systems that have a different orientation in 3D space [28, 29]. Other implications are that although some of the included studies did report ‘normal values’ for 3D alignment parameters, these ‘normal values’ are only valid within the context of that particular study and cannot be broadly adopted; and that the normal alignment parameter values of the gold standard 2D framework for leg alignment analysis cannot be blindly adopted in 3D, as this might lead to an inappropriate correction in case of an osteotomy around the knee in clinical practice. Therefore, the 3D workflow is a promising improvement over the current gold standard 2D workflow, but a universal 3D framework for leg alignment analysis is necessary.

Despite the high variability, several underlying principles to the four groups in which the reported methods were categorized can be identified: (1) joint orientation of flatter articular surfaces might be acquired by a calculated plane through marked articular surface data (Figure 4a2); (2) joint orientation of rounder articular surfaces might be acquired by a best fit of a geometrical shape to marked articular surface data (Figure 3b1); (3) joint centres and mechanical axes might be acquired with centroids of marked articular surface data (e.g., the midpoint between acquired centroids of the marked posterior medial and lateral femoral condyles; Figure 3b2); and (4) non‐linear femoral and tibial anatomical axes might be acquired by a calculated line from cortical or endosteal bone data (Figure 5d). One other identified principle might be fundamental to all of the ones mentioned above: the selection of all available relevant surface data on a 3D bone model. In contrast to the current gold standard 2D framework and some of the reported 3D frameworks for leg alignment analysis, which use one single or a few data point(s) only to derive landmarks for basic line drawing, this principle uses all available relevant surface data of the 3D bone model in order to derive axes and joint orientations for alignment parameters and coordinate systems. However, considering the results of the present study, these principles are not yet universally adopted. Therefore, the authors propose that these principles might be used as the foundation for a higher level of agreement or even consensus on a universal future 3D framework for leg alignment analysis.

Interestingly, although for the purpose of reference, alignment parameters are expressed in the coronal, sagittal or axial anatomical planes [81], only a third of included studies that reported on knee‐related leg alignment parameters also reported a complete coordinate system (and their resulting anatomical reference planes). Using a complete coordinate system in the context of alignment analysis and osteotomy planning is important to identify and control the resultant effect of an osteotomy on alignment in all three anatomical reference planes simultaneously [40, 103]. The gold standard to establish 3D joint coordinate systems with anatomical reference planes stems from the International Society of Biomechanics' (ISB) recommendation on definitions of joint coordinate systems’ [108]. As per ISB recommendations, indeed, the included papers that reported complete coordinate systems most frequently established a femoral and tibial coordinate system based on their mechanical axis and a central medial‐lateral axis (FCA and the PTJ ML, respectively). Still, a relevant minority of 15% of the included papers reported complete tibial coordinate systems based on the longitudinal axis on the normal vector to the best‐fit plane of (the articular surface of) the tibial plateau. However, the tibial plateau is tilted (and not orthogonal) relative to the mTA, and in both the context of osteotomies around the knee and total knee arthroplasties, leg alignment analysis seems most appropriate when it is based on mechanical axes [26, 67]. Therefore, the authors propose that the coordinate systems (in which anatomical planes for alignment parameters of the leg are expressed) be based on mechanical axes and central medial‐lateral axes, in addition to the principles described above.

Furthermore, for the central medial‐lateral axis of the femoral coordinate system, the ISB recommends using a femoral epicondylar axis. This axis is based on two arbitrary landmark points, instead of an FCA (as per findings of the present study) that is based on the articular surface data of the femoral condyles [108]. The same goes for the central medial‐lateral tibial axis of the tibial coordinate system, where the ISB recommends the line connecting two arbitrary landmark points (‘most medial point’ of the medial tibial plateau and the ‘most lateral point’ of the lateral tibial plateau) [108]. In these two instances, the principle of using all available relevant surface data of the 3D bone model in order to derive axes and joint orientations is not met. Therefore, the authors propose to define the medial‐lateral orientation of a femoral and tibial coordinate system according to the above‐described principles, based on all available articular surface data instead of single data points.

To our knowledge, this is the first systematic review investigating the reported methods on how to derive axes and joint orientations of 3D bone models for knee‐related leg alignment parameters and coordinate systems for the femur, tibia, and leg. Nevertheless, in addition to the limitations inherent to all systematic reviews, this study has several limitations. First, this review focused on CT‐based 3D bone models, thereby excluding studies reporting MRI or the EOS 2D/3D imaging system. However, although interest in MRI‐ and EOS‐based 3D models is increasing, to date, CT is the preferred imaging modality for segmentation purposes. Second, this study focused on native knees, while arthroplasty‐related studies show increasing interest in 3D knee‐related leg alignment analysis and the optimal positioning of arthroplasty components. Thus, methods to derive axes and joint orientations from 3D bone models for alignment parameters and coordinate systems might have been missed. However, as methods in arthroplasty‐related studies might not be applicable for correction osteotomies due to the absence of osseous landmark points, only relevant 3D methods that could be performed on a native knee were included in this systematic review. Third, only papers reporting knee‐related leg alignment or coordinate systems that were, ultimately, reported as lines were included. Thus, papers reporting on other methods that define leg alignment parameters might have been missed. However, even studies that used all available relevant data to define joint orientation ultimately reported this joint orientation as a line (e.g., best‐fit plane through all data points of the tibial plateau defines the proximal tibial joint orientation, and the intersecting line of the joint orientation plane with the anatomical coronal and sagittal plane is used to calculate the joint orientation angle). Irrespective of these limitations, the results of this study could spark the community to make progress in this particular area of the field.

The clinical relevance of this work is, first, that it reveals a high variability of reported methods in the field of 3D knee‐related leg alignment analysis. This indicates that generalizable 3D reference values for normal alignment parameters are missing, and comparability between alignment parameter values reported in different studies is impossible. Second, this work identifies underlying principles that might lead to agreement on a universal framework for 3D leg alignment analysis. Considering the increasing popularity of 3D use in a clinical setting, one would expect such a universal framework to be a prerequisite, as is the case for the 2D framework.

## CONCLUSION

There is high variability in 3D knee‐related leg alignment analysis. Therefore, universal 3D reference values for alignment parameters cannot yet be defined, and comparison of alignment parameter values between different studies is impossible. However, several underlying principles to the reported methods were identified, which could serve to reach more agreement on a future universal 3D framework for leg alignment analysis.

## AUTHOR CONTRIBUTIONS

Romy M. ten Heggeler, Roy A. G. Hoogeslag and Feike de Graaff all made substantial contributions to the conception and design of the study. Romy M. ten Heggeler, Roy A.G. Hoogeslag and Quinten W. T. Veerman have made substantial contributions to the extraction of data. All authors have made substantial contributions to the analysis and interpretation of data and have been involved in drafting the manuscript and revising it critically for important intellectual content.

## CONFLICT OF INTEREST STATEMENT

The authors declare no conflict of interest.

## ETHICS STATEMENT

Ethical approval was not required for this systematic review.

## Supporting information

Supporting information.

## Data Availability

All data are made available in Supporting Information S1.

## References

[1] Adachi, T. , Kato, Y. , Kiyotomo, D. , Kawamukai, K. , Takazawa, S. , Suzuki, T. et al. (2023) Accuracy verification of four‐dimensional CT analysis of knee joint movements: a pilot study using a knee joint model and motion‐capture system. Cureus, 15, e35616. Available from: 10.7759/cureus.35616 37007305 PMC10065360

[2] Amirtharaj, M.J. , Hardy, B.M. , Kent, R.N. , Nawabi, D.H. , Wickiewicz, T.L. , Pearle, A.D. et al. (2018) Automated, accurate, and three‐dimensional method for calculating sagittal slope of the tibial plateau. Journal of Biomechanics, 79, 212–217. Available from: 10.1016/J.JBIOMECH.2018.07.047 30217556

[3] An, H.M. , Wen, J.X. , Gu, W. , Chen, J.Y. , Chai, W. & Li, R. (2024) Discrepancies in sagittal alignment of the lower extremity among different brands of robotic total knee arthroplasty systems. The Journal of Arthroplasty, 39, 2248–2253. Available from: 10.1016/j.arth.2024.03.029 38508345

[4] Arn Roth, T. , Jokeit, M. , Sutter, R. , Vlachopoulos, L. , Fucentese, S.F. , Carrillo, F. et al. (2024) Deep‐learning based 3D reconstruction of lower limb bones from biplanar radiographs for preoperative osteotomy planning. International Journal of Computer Assisted Radiology and Surgery, 19, 1843–1853. Available from: 10.1007/s11548-024-03110-5 38573567 PMC11365828

[5] Berryman, F. , Pynsent, P. & McBryde, C. (2014) A semi‐automated method for measuring femoral shape to derive version and its comparison with existing methods. International Journal for Numerical Methods in Biomedical Engineering, 30, 1314–1325. Available from: 10.1002/CNM.2659 25044860

[6] Brunner, J. , Jörgens, M. , Weigert, M. , Kümpel, H. , Degen, N. & Fuermetz, J. (2023) Significant changes in lower limb alignment due to flexion and rotation—a systematic 3D simulation of radiographic measurements. Knee Surgery, Sports Traumatology, Arthroscopy, 31, 1483–1490. Available from: 10.1007/s00167-022-07302-x PMC10050026 36595052

[7] Chalmers, B.P. , Borsinger, T.M. , Quevedo Gonzalez, F.J. , Vigdorchik, J.M. , Haas, S.B. & Ast, M.P. (2023) Referencing the center of the femoral head during robotic or computer‐navigated primary total knee arthroplasty results in less femoral component flexion than the traditional intramedullary axis. The Knee, 44, 172–179. Available from: 10.1016/j.knee.2023.08.006 37672908

[8] Chalmers, B.P. , Quevedo‐Gonzalez, F. , Gausden, E.B. , Jerabek, S.A. , Haas, S.B. & Ast, M.P. (2022) Posterior tibial slope in computer‐navigated total knee arthroplasty: the transmalleolar sagittal axis underestimates slope compared to traditional intramedullary Axis. The Journal of Arthroplasty, 37, S207–S210. Available from: 10.1016/j.arth.2022.02.085 35240280

[9] Cho, B.W. , Lee, T.‐H. , Kim, S. , Choi, C.‐H. , Jung, M. , Lee, K.Y. et al. (2021) Evaluation of the reliability of lower extremity alignment measurements using EOS imaging system while standing in an even weight‐bearing posture. Scientific Reports, 11, 2203922039. Available from: 10.1038/s41598-021-01646-z PMC8585885 34764394

[10] Cho, H.J. , Kwak, D.S. & Kim, I.B. (2015) Morphometric evaluation of Korean femurs by geometric computation: comparisons of the sex and the population. BioMed Research International, 2015, 1–9. Available from: 10.1155/2015/730538 PMC4564606 26413540

[11] Chung, J.H. , Choi, C.H. , Kim, S.H. , Kim, S.J. , Suk, Y.J. & Jung, M. (2022) Effect of the sagittal osteotomy inclination angle on the posterior tibial slope change in high tibial osteotomy: three‐dimensional simulation study. Scientific Reports, 12, 19254. Available from: 10.1038/s41598-022-23412-5 36357467 PMC9649806

[12] Citak, M. , Oszwald, M. , O'Loughlin, P.F. , Citak, M. , Kendoff, D. , Hüfner, T. et al. (2010) Three‐dimensional measurement of femoral antetorsion: comparison to a conventional radiological method. Archives of Orthopaedic and Trauma Surgery, 130, 513–518. Available from: 10.1007/S00402-009-0923-8 19568758

[13] De Valk, E.J. , Noorduyn, J.C.A. & Mutsaerts, E.L.A.R. (2016) How to assess femoral and tibial component rotation after total knee arthroplasty with computed tomography: a systematic review. Knee Surgery, Sports Traumatology, Arthroscopy, 24, 3517–3528. Available from: 10.1007/S00167-016-4325-5 27655141

[14] Dean, R.S. , DePhillipo, N.N. , Chahla, J. , Larson, C.M. & LaPrade, R.F. (2021) Posterior tibial slope measurements using the anatomic axis are significantly increased compared with those that use the mechanical axis. Arthroscopy: The Journal of Arthroscopic & Related Surgery, 37, 243–249. Available from: 10.1016/J.ARTHRO.2020.09.006 32949632

[15] Degen, N. , Sass, J. , Jalali, J. , Kovacs, L. , Euler, E. , Prall, W.C. et al. (2020) Three‐dimensional assessment of lower limb alignment: reference values and sex‐related differences. The Knee, 27, 428–435. Available from: 10.1016/J.KNEE.2019.11.009 31806504

[16] Eckhoff, D. , Hogan, C. , DiMatteo, L. , Robinson, M. & Bach, J. (2007) AN ABJS BEST PAPER: difference between the epicondylar and cylindrical axis of the knee. Clinical Orthopaedics & Related Research, 461, 238–244. Available from: 10.1097/BLO.0B013E318112416B 17549027

[17] Ehlinger, M. , Favreau, H. , Murgier, J. & Ollivier, M. (2023) Knee osteotomies: the time has come for 3D planning and patient‐specific instrumentation. Orthopaedics & Traumatology: Surgery & Research, 109, 103611. Available from: 10.1016/j.otsr.2023.103611 36972897

[18] Enomoto, H. , Nakamura, T. , Waseda, A. , Niki, Y. , Toyama, Y. & Suda, Y. (2013) A novel and reproducible reference axis for distal tibial axial rotation. The Journal of Arthroplasty, 28, 788–791. Available from: 10.1016/j.arth.2012.11.005 23489723

[19] Factor, S. , Gurel, R. , Dan, D. , Benkovich, G. , Sagi, A. , Abialevich, A. et al. (2024) Validating a novel 2D to 3D knee reconstruction method on preoperative total knee arthroplasty patient anatomies. Journal of Clinical Medicine, 13, 1255. Available from: 10.3390/jcm13051255 38592666 PMC10931545

[20] Fischer, M.C.M. , Grothues, S.A.G.A. , Habor, J. , de la Fuente, M. & Radermacher, K. (2020) A robust method for automatic identification of femoral landmarks, axes, planes and bone coordinate systems using surface models. Scientific Reports, 10, 20859. Available from: 10.1038/s41598-020-77479-z 33257714 PMC7704624

[21] Flury, A. , Hodel, S. , Hasler, J. , Hooman, E. , Fucentese, S.F. & Vlachopoulos, L. (2022) The winking sign is an indicator for increased femorotibial rotation in patients with recurrent patellar instability. Knee Surgery, Sports Traumatology, Arthroscopy, 30, 3651–3658. Available from: 10.1007/s00167-022-06971-y PMC9568440 35438307

[22] Fürmetz, J. , Sass, J. , Ferreira, T. , Jalali, J. , Kovacs, L. , Mück, F. et al. (2019) Three‐dimensional assessment of lower limb alignment: accuracy and reliability. The Knee, 26, 185–193. Available from: 10.1016/J.KNEE.2018.10.011 30473372

[23] Hanada, M. , Hotta, K. & Matsuyama, Y. (2020) A computer simulation study for preserving the tibial posterior slope in open‐wedge high tibial osteotomy. European Journal of Orthopaedic Surgery & Traumatology, 30, 1285–1291. Available from: 10.1007/S00590-020-02703-5 32430728

[24] Hancock, C.W. , Winston, M.J. , Bach, J.M. , Davidson, B.S. & Eckhoff, D.G. (2013) Cylindrical axis, not epicondyles, approximates perpendicular to knee axes. Clinical Orthopaedics & Related Research, 471, 2278–2283. Available from: 10.1007/S11999-013-2864-3 23536175 PMC3676579

[25] Hartel, M.J. , Petersik, A. , Schmidt, A. , Kendoff, D. , Nüchtern, J. , Rueger, J.M. et al. (2016) Determination of femoral neck angle and torsion angle utilizing a novel three‐dimensional modeling and analytical technology based on CT datasets. PLoS One, 11, e0149480. Available from: 10.1371/JOURNAL.PONE.0149480 26933877 PMC4775021

[26] Hirschmann, M.T. , Moser, L.B. , Amsler, F. , Behrend, H. , Leclerq, V. & Hess, S. (2019) Functional knee phenotypes: a novel classification for phenotyping the coronal lower limb alignment based on the native alignment in young non‐osteoarthritic patients. Knee Surgery, Sports Traumatology, Arthroscopy, 27, 1394–1402. Available from: 10.1007/s00167-019-05509-z 30976825

[27] Hirschmann, M.T. , von Eisenhart‐Rothe, R. & Graichen, H. (2023) Any technology assisting total knee arthroplasty (TKA) will fail without the correct 3D alignment and balancing target. Knee Surgery, Sports Traumatology, Arthroscopy, 31, 733–735. Available from: 10.1007/s00167-023-07345-8 36800007

[28] Ho, J.P.Y. , Merican, A.M. , Ayob, K.A. , Sulaiman, S.H. & Hashim, M.S. (2021) Tibia vara in Asians: myth or fact? Verification with three‐dimensional computed tomography. Journal of Orthopaedic Surgery (Hong Kong), 29, 2309499021992618. Available from: 10.1177/2309499021992618 33632009

[29] Ho, J.P.Y. , Merican, A.M. , Hashim, M.S. , Abbas, A.A. , Chan, C.K. & Mohamad, J.A. (2017) Three‐dimensional computed tomography analysis of the posterior tibial slope in 100 knees. The Journal of Arthroplasty, 32, 3176–3183. Available from: 10.1016/J.ARTH.2017.04.060 28579444

[30] Hoch, A. , Hasler, J. , Schenk, P. , Ackermann, J. , Ebert, L. , Fürnstahl, P. et al. (2022) Registration based assessment of femoral torsion for rotational osteotomies based on the contralateral anatomy. BMC Musculoskeletal Disorders, 23, 962. Available from: 10.1186/s12891-022-05941-2 36348364 PMC9641797

[31] Hoch, A. , Jud, L. , Roth, T. , Vlachopoulos, L. , Fürnstahl, P. & Fucentese, S.F. (2020) A real 3D measurement technique for the tibial slope: differentiation between different articular surfaces and comparison to radiographic slope measurement. BMC Musculoskeletal Disorders, 21, 635. Available from: 10.1186/S12891-020-03657-9 32979919 PMC7520019

[32] Hodel, S. , Arn‐Roth, T. , Haug, F. , Carillo, F. , Vlachopoulos, L. , Fucentese, S.F. et al. (2024) The influence of the weight‐bearing state on three‐dimensional (3D) planning in lower extremity realignment—analysis of novel vs. state‐of‐the‐art planning approaches. Archives of Orthopaedic and Trauma Surgery, 144, 1989–1996. Available from: 10.1007/s00402-024-05289-3 38554205 PMC11093806

[33] Hodel, S. , Hasler, J. , Roth, T.A. , Flury, A. , Sutter, C. , Fucentese, S.F. et al. (2024) Validation of a three‐dimensional weight‐bearing measurement protocol for medial open‐wedge high tibial osteotomy. Journal of Clinical Medicine, 13, 1280. Available from: 10.3390/jcm13051280 38592100 PMC10931564

[34] Howick, J. , Chalmers, I. , Glasziou, P. , Greenhalgh, T. , Heneghan, C. & Liberati, A. et al. (2011) The Oxford Levels of Evidence 2. Oxford Centre for Evidence‐Based Medicine. https://www.cebm.ox.ac.uk/resources/levels-of-evidence/ocebm-levels-of-evidence. [Accessed 5th October 2021].

[35] Huan, W. , Mochizuki, T. , Tanifuji, O. & Kawashima, H. (2023) Variability of functional knee phenotype for coronal alignment in advanced varus knee osteoarthritis in the Japanese population. Knee Surgery, Sports Traumatology, Arthroscopy, 31, 1451–1461. Available from: 10.1007/s00167-022-07248-0 36449045

[36] Ikuta, F. , Yoneta, K. , Miyaji, T. , Kidera, K. , Yonekura, A. , Osaki, M. et al. (2020) Association between stages of medial compartment osteoarthritis and three‐dimensional knee alignment in the supine position: a cross‐sectional study. Journal of Clinical Orthopaedics and Trauma, 11, S130–S136. Available from: 10.1016/J.JCOT.2019.10.011 31992933 PMC6978193

[37] Jacquet, C. , Laumonerie, P. , LiArno, S. , Faizan, A. , Sharma, A. , Dagneaux, L. et al. (2019) Contralateral preoperative templating of lower limbs' mechanical angles is a reasonable option. Knee Surgery, Sports Traumatology, Arthroscopy, 28, 1445–1451. Available from: 10.1007/S00167-019-05524-0 31073842

[38] Jamali, A.A. , Meehan, J.P. , Moroski, N.M. , Anderson, M.J. , Lamba, R. & Parise, C. (2017) Do small changes in rotation affect measurements of lower extremity limb alignment? Journal of Orthopaedic Surgery and Research, 12, 77. Available from: 10.1186/S13018-017-0571-6 28532505 PMC5441094

[39] Jörgens, M. , Keppler, A.M. , Ahrens, P. , Prall, W.C. , Bergstraesser, M. , Bachmeier, A.T. et al. (2024) 3D osteotomies—improved accuracy with patient‐specific instruments (PSI). European Journal of Trauma and Emergency Surgery, 50, 3–10. Available from: 10.1007/s00068-022-02060-4 35879618 PMC10923740

[40] Jörgens, M. , Keppler, A.M. , Degen, N. , Bachmeier, A.T. , Bergstraesser, M. , Sass, J. et al. (2022) Reliability of 3D planning and simulations of medial open wedge high tibial osteotomies. Journal of Orthopaedic Surgery (Hong Kong), 30, 10225536221101699. Available from: 10.1177/10225536221101699 35694778

[41] Jud, L. , Roth, T. , Fürnstahl, P. , Vlachopoulos, L. , Sutter, R. & Fucentese, S.F. (2020) The impact of limb loading and the measurement modality (2D versus 3D) on the measurement of the limb loading dependent lower extremity parameters. BMC Musculoskeletal Disorders, 21, 1–9. Available from: 10.1186/S12891-020-03449-1 PMC7329436 32605616

[42] Jud, L. , Singh, S. , Tondelli, T. , Fürnstahl, P. , Fucentese, S.F. & Vlachopoulos, L. (2020) Combined correction of tibial torsion and tibial tuberosity‐trochlear groove distance by supratuberositary torsional osteotomy of the tibia. The American Journal of Sports Medicine, 48, 2260–2267. Available from: 10.1177/0363546520929687 32551826

[43] Jud, L. , Trache, T. , Tondelli, T. , Fürnstahl, P. , Fucentese, S.F. & Vlachopoulos, L. (2019) Rotation or flexion alters mechanical leg axis measurements comparably in patients with different coronal alignment. Knee Surgery, Sports Traumatology, Arthroscopy, 28, 3128–3134. Available from: 10.1007/S00167-019-05779-7 31705148

[44] Jung, S.H. , Jung, M. , Chung, K. , Kim, S. , Park, J. , Lee, J.H. et al. (2024) Factors causing unintended sagittal and axial alignment changes in high tibial osteotomy: comparative 3‐dimensional analysis of simulation and actual surgery. The American Journal of Sports Medicine, 52, 1543–1553. Available from: 10.1177/03635465241241539 38616541

[45] Kai, S. , Sato, T. , Koga, Y. , Omori, G. , Kobayashi, K. , Sakamoto, M. et al. (2014) Automatic construction of an anatomical coordinate system for three‐dimensional bone models of the lower extremities—pelvis, femur, and tibia. Journal of Biomechanics, 47, 1229–1233. Available from: 10.1016/J.JBIOMECH.2013.12.013 24456665

[46] Karasavvidis, T. , Pagan Moldenhauer, C.A. , Lustig, S. , Vigdorchik, J.M. & Hirschmann, M.T. (2023) Definitions and consequences of current alignment techniques and phenotypes in total knee arthroplasty (TKA)—there is no winner yet. Journal of Experimental Orthopaedics, 10, 120. Available from: 10.1186/s40634-023-00697-7 37991599 PMC10665290

[47] Kawahara, S. , Hara, D. , Murakami, K. , Hamai, S. , Akasaki, Y. , Tsushima, H. et al. (2022) Smaller femoral neck anteversion in varus knees than in healthy and valgus knees. Clinical Anatomy, 35, 1044–1050. Available from: 10.1002/ca.23862 35333417

[48] Kim, J.H. , Kim, H.Y. & Lee, D.H. (2020) Opening gap width influences distal tibial rotation below the osteotomy site following open wedge high tibial osteotomy. PLoS One, 15, e0227969. Available from: 10.1371/JOURNAL.PONE.0227969 31945112 PMC6964860

[49] Kuiper, R.J.A. , Seevinck, P.R. , Viergever, M.A. , Weinans, H. & Sakkers, R.J.B. (2023) Automatic assessment of lower‐limb alignment from computed tomography. The Journal of Bone and Joint Surgery. American Volume, 105, 700–712. Available from: 10.2106/jbjs.22.00890 36947661

[50] Lee, B.H. , Ha, C.W. , Moon, S.W. , Chang, M. , Kim, H.Y. , Park, S.H. et al. (2017) Three‐dimensional relationships between secondary changes and selective osteotomy parameters for biplane medial open‐wedge high tibial osteotomy. The Knee, 24, 362–371. Available from: 10.1016/J.KNEE.2016.11.010 28169100

[51] Lei, K. , Liu, L.M. , Luo, J.M. , Ma, C. , Feng, Q. , Yang, L. et al. (2022) Could surgical transepicondylar axis be identified accurately in preoperative 3D planning for total knee arthroplasty? A reproducibility study based on 3D‐CT. Arthroplasty, 4, 46. Available from: 10.1186/s42836-022-00147-2 36244969 PMC9575283

[52] Lei, K. , Liu, L.M. , Xiang, Y. , Chen, X. , Fan, H.Q. , Peng, Y. et al. (2020) Clinical value of CT‐based patient‐specific 3D preoperative design combined with conventional instruments in primary total knee arthroplasty: a propensity score‐matched analysis. Journal of Orthopaedic Surgery and Research, 15, 591. Available from: 10.1186/s13018-020-02123-5 33298106 PMC7724895

[53] Lenchik, L. , Heacock, L. , Weaver, A.A. , Boutin, R.D. , Cook, T.S. , Itri, J. et al. (2019) Automated segmentation of tissues using CT and MRI: a systematic review. Academic Radiology, 26, 1695–1706. Available from: 10.1016/J.ACRA.2019.07.006 31405724 PMC6878163

[54] León‐Muñoz, V.J. , López‐López, M. , Martínez‐Martínez, F. & Santonja‐Medina, F. (2020) Comparison of weight‐bearing full‐length radiographs and computed‐tomography‐scan‐based three‐dimensional models in the assessment of knee joint coronal alignment. The Knee, 27, 543–551. Available from: 10.1016/J.KNEE.2019.11.017 31954608

[55] León‐Muñoz, V.J. , Manca, S. , López‐López, M. , Martínez‐Martínez, F. & Santonja‐Medina, F. (2021) Coronal and axial alignment relationship in Caucasian patients with osteoarthritis of the knee. Scientific Reports, 11, 7836. Available from: 10.1038/s41598-021-87483-6 33837279 PMC8035173

[56] León‐Muñoz, V.J. , Parrinello, A. , Galloni, G. , Lisón‐Almagro, A.J. , López‐López, M. , Martínez‐Martínez, F. et al. (2022) Reliability of the posterior condylar offset. Journal of Orthopaedic Research, 40, 1794–1800. Available from: 10.1002/jor.25205 34717014

[57] Li, C. , Chen, J. , Yang, Y. , Jin, Y. , Wang, C. , Tsai, T.Y. et al. (2022) Asymmetry of posterior condyles in resection plane and axial curvature for total knee arthroplasty. Orthopaedic Surgery, 14, 3340–3348. Available from: 10.1111/os.13529 36346140 PMC9732622

[58] Li, Z. , Liu, G. , Tian, R. , Kong, N. , Li, Y. , Li, Y. et al. (2021) The patellofemoral morphology and the normal predicted value of tibial tuberosity‐trochlear groove distance in the Chinese population. BMC Musculoskeletal Disorders, 22, 1–13. Available from: 10.1186/S12891-021-04454-8/TABLES/3 34162383 PMC8223279

[59] Liu, K. , Liu, X. , Guan, Y. , Ma, H. , Fu, D. & Fan, Z. (2023) Accuracy and reproducibility analysis of different reference axes for femoral prosthesis rotation alignment in TKA based on 3D CT femoral model. BMC Musculoskeletal Disorders, 24, 660. Available from: 10.1186/s12891-023-06781-4 37596664 PMC10439596

[60] Liu, K. , Liu, Y. , Fan, Z. & Fu, D. (2023) Accuracy and reproducibility of two‐dimensional computed tomography‐based positioning of femoral component rotational alignment in preoperative planning for total knee arthroplasty. Journal of Orthopaedic Surgery and Research, 18, 964. Available from: 10.1186/s13018-023-04466-1 38098082 PMC10722822

[61] Liu, L. , Lei, K. , Chen, X. , Fan, H. , Yang, L. & Guo, L. (2022) Is valgus cut angle based on radiographic measurements in total knee arthroplasty really inaccurate? A comparison of two‐ and three‐dimensional measurements. The Journal of Knee Surgery, 35, 1563–1570. Available from: 10.1055/s-0041-1728785 33915574

[62] Liu, L. , Lei, K. , Du, D. , Lin, Y. , Pan, Z. & Guo, L. (2024) Functional knee phenotypes appear to be more suitable for the Chinese OA population compared with CPAK classification: a study based on 3D CT reconstruction models. Knee Surgery, Sports Traumatology, Arthroscopy, 32, 1264–1274. Available from: 10.1002/ksa.12130 38488258

[63] Liu, L. , Lei, K. , Guo, L. , Chen, X. , Yang, P. , Fu, D. et al. (2022) Surgical transepicondylar axis is not a reliable reference when there was lateral femoral bowing. Orthopaedic Surgery, 14, 3209–3215. Available from: 10.1111/os.13545 36250537 PMC9732584

[64] Liu, L.M. , Lei, K. , Chen, X. , Fu, D.J. , Yang, P. , Yang, L. et al. (2023) Proximal external femoral torsion increases lateral femoral shaft bowing: a study based on 3D CT reconstruction models. Knee Surgery, Sports Traumatology, Arthroscopy, 31, 1524–1532. Available from: 10.1007/S00167-021-06753-Y 34609540

[65] Luís, N.M. & Varatojo, R. (2021) Radiological assessment of lower limb alignment. EFORT Open Reviews, 6, 487–494. Available from: 10.1302/2058-5241.6.210015 34267938 PMC8246117

[66] Ma, Q.L. , Lipman, J.D. , Cheng, C.K. , Wang, X.N. , Zhang, Y.Y. & You, B. (2017) A comparison between chinese and caucasian 3‐dimensional bony morphometry in presimulated and postsimulated osteotomy for total knee arthroplasty. The Journal of Arthroplasty, 32, 2878–2886. Available from: 10.1016/j.arth.2017.03.069 28457760

[67] MacDessi, S.J. , Griffiths‐Jones, W. , Harris, I.A. , Bellemans, J. & Chen, D.B. (2021) Coronal Plane Alignment of the Knee (CPAK) classification. The Bone & Joint Journal, 103‐B, 329–337. Available from: 10.1302/0301-620X.103B2.BJJ-2020-1050.R1 PMC7954147 33517740

[68] Meier, M. , Janssen, D. , Koeck, F.X. , Thienpont, E. , Beckmann, J. & Best, R. (2020) Variations in medial and lateral slope and medial proximal tibial angle. Knee Surgery, Sports Traumatology, Arthroscopy, 29(3), 939–946. Available from: 10.1007/S00167-020-06052-Y 32390118

[69] Meric, G. , Gracitelli, G.C. , Aram, L.J. , Swank, M.L. & Bugbee, W.D. (2015) Variability in distal femoral anatomy in patients undergoing total knee arthroplasty: measurements on 13,546 computed tomography scans. The Journal of Arthroplasty, 30, 1835–1838. Available from: 10.1016/j.arth.2015.04.024 26021904

[70] Micicoi, G. , Jacquet, C. , Sharma, A. , LiArno, S. , Faizan, A. , Kley, K. et al. (2020) Neutral alignment resulting from tibial vara and opposite femoral valgus is the main morphologic pattern in healthy middle‐aged patients: an exploration of a 3D‐CT database. Knee Surgery, Sports Traumatology, Arthroscopy, 29(3), 849–858. Available from: 10.1007/S00167-020-06030-4 32372282

[71] Miranda, D.L. , Rainbow, M.J. , Leventhal, E.L. , Crisco, J.J. & Fleming, B.C. (2010) Automatic determination of anatomical coordinate systems for three‐dimensional bone models of the isolated human knee. Journal of Biomechanics, 43, 1623–1626. Available from: 10.1016/J.JBIOMECH.2010.01.036 20167324 PMC2866785

[72] Modenese, L. & Renault, J.B. (2021) Automatic generation of personalised skeletal models of the lower limb from three‐dimensional bone geometries. Journal of Biomechanics, 116, 110186110186. Available from: 10.1016/J.JBIOMECH.2020.110186 33515872

[73] Moon, H.‐S. , Choi, C.‐H. , Jung, M. , Lee, D.‐Y. , Kim, J.‐H. & Kim, S.‐H. (2020) The effect of knee joint rotation in the sagittal and axial plane on the measurement accuracy of coronal alignment of the lower limb. BMC Musculoskeletal Disorders, 21, 470. Available from: 10.1186/S12891-020-03487-9 32680484 PMC7368736

[74] Moon, S.W. , Park, S.H. , Lee, B.H. , Oh, M. , Chang, M. , Ahn, J.H. et al. (2015) The effect of hinge position on posterior tibial slope in medial open‐wedge high tibial osteotomy. Arthroscopy: The Journal of Arthroscopic & Related Surgery, 31, 1128–1133. Available from: 10.1016/J.ARTHRO.2015.01.009 25744929

[75] Nedopil, A.J. , Hernandez, A.M. , Boone, J.M. , Howell, S.M. & Hull, M.L. (2023) Correcting for distal femoral asymmetry is necessary to determine postoperative alignment deviations from planned alignment of the femoral component. The Knee, 42, 193–199. Available from: 10.1016/j.knee.2023.01.013 37054496

[76] Nguyen, H.C. , Gielis, W.P. , van Egmond, N. , Weinans, H. , Slump, C.H. , Sakkers, R.J.B. et al. (2021) The need for a standardized whole leg radiograph guideline: the effects of knee flexion, leg rotation, and X‐ray beam height. Journal of Cartilage & Joint Preservation, 1, 100022100022. Available from: 10.1016/J.JCJP.2021.100022

[77] NHLBI . (n.d.). Study Quality Assessment Tools. https://www.nhlbi.nih.gov/health-topics/study-quality-assessment-tools [Accessed 5th October 2021].

[78] Ohmori, T. , Kabata, T. , Kajino, Y. , Inoue, D. , Ueno, T. , Taga, T. et al. (2022) Importance of three‐dimensional evaluation of surgical transepicondylar axis in total knee arthroplasty. The Journal of Knee Surgery, 35, 32–38. Available from: 10.1055/s-0040-1712087 32512597

[79] Okamoto, S. , Mizu‐uchi, H. , Okazaki, K. , Hamai, S. , Tashiro, Y. , Nakahara, H. et al. (2016) Two‐dimensional planning can result in internal rotation of the femoral component in total knee arthroplasty. Knee Surgery, Sports Traumatology, Arthroscopy, 24, 229–235. Available from: 10.1007/s00167-014-3370-1 25297705

[80] Ouzzani, M. , Hammady, H. , Fedorowicz, Z. & Elmagarmid, A. (2016) Rayyan—a web and mobile app for systematic reviews. Systematic Reviews, 5, 210. Available from: 10.1186/s13643-016-0384-4 27919275 PMC5139140

[81] Paley, D. (2002) Principles of Deformity correction. Berlin Heidelberg New York: Springer‐Verlag.

[82] Paley, D. , Herzenberg, J.E. , Tetsworth, K. , McKie, J. & Bhave, A. (1994) Deformity planning for frontal and sagittal plane corrective osteotomies. Orthopedic Clinics of North America, 25(3), 425–465. Available from: 10.1016/S0030-5898(20)31927-1 8028886

[83] Pangaud, C. , Laumonerie, P. , Dagneaux, L. , LiArno, S. , Wellings, P. , Faizan, A. et al. (2020) Measurement of the posterior tibial slope depends on ethnicity, sex, and lower limb alignment: a computed tomography analysis of 378 healthy participants. Orthopaedic Journal of Sports Medicine, 8, 2325967119895258. Available from: 10.1177/2325967119895258 32047827 PMC6984458

[84] Preston, B. , Harris, S. , Villet, L. , Mattathil, C. , Cobb, J. & Rivière, C. (2022) The medial condylar wall is a reliable landmark to kinematically align the femoral component in medial UKA: an in‐silico study. Knee Surgery, Sports Traumatology, Arthroscopy, 30, 3220–3227. Available from: 10.1007/s00167-021-06683-9 PMC9418071 34363490

[85] Qin, J. , Chen, D. , Xu, Z. , Shi, D. , Dai, J. & Jiang, Q. (2018) Evaluation of the effect of the sulcus angle and lateral to medial facet ratio of the patellar groove on patella tracking in aging subjects with stable knee joint. BioMed Research International, 2018, 1–5. Available from: 10.1155/2018/4396139 PMC5964416 29854753

[86] Renault, J.B. , Aüllo‐Rasser, G. , Donnez, M. , Parratte, S. & Chabrand, P. (2018) Articular‐surface‐based automatic anatomical coordinate systems for the knee bones. Journal of Biomechanics, 80, 171–178. Available from: 10.1016/J.JBIOMECH.2018.08.028 30213649

[87] Roth, T. , Carrillo, F. , Wieczorek, M. , Ceschi, G. , Esfandiari, H. , Sutter, R. et al. (2021) Three‐dimensional preoperative planning in the weight‐bearing state: validation and clinical evaluation. Insights into Imaging, 12, 44. Available from: 10.1186/s13244-021-00994-8 33825985 PMC8026795

[88] Roth, T. , Sigrist, B. , Wieczorek, M. , Schilling, N. , Hodel, S. , Walker, J. et al. (2023) An automated optimization pipeline for clinical‐grade computer‐assisted planning of high tibial osteotomies under consideration of weight‐bearing. Computer Assisted Surgery, 28, 2211728. Available from: 10.1080/24699322.2023.2211728 37191179

[89] Sadoghi, P. , Hirschmann, M.T. , Karlsson, J. & Klasan, A. (2024) The neglected factor of constitutional sagittal alignment and its implications for total knee arthroplasty. Knee Surgery, Sports Traumatology, Arthroscopy, 32, 10–12. Available from: 10.1002/ksa.12013 38226765

[90] Sasaki, R. , Niki, Y. , Kaneda, K. , Yamada, Y. , Nagura, T. , Nakamura, M. et al. (2023) Three‐dimensional joint surface orientation does not correlate with two‐dimensional coronal joint line orientation in knee osteoarthritis: three‐dimensional analysis of upright computed tomography. The Knee, 43, 10–17. Available from: 10.1016/j.knee.2023.05.001 37207557

[91] Sato, A. , Takagi, H. , Koya, T. , Espinoza Orías, A.A. , Kanzaki, K. & Inoue, N. (2023) Clinical three‐dimensional anatomy of the femur considering navigation‐aided surgery of total knee arthroplasty in Japanese patients. The Knee, 41, 214–220. Available from: 10.1016/j.knee.2022.12.001 36724580

[92] Sava, M.P. , Leica, A. , Amsler, F. , Leles, S. & Hirschmann, M.T. (2024) Only 26% of native knees show an identical coronal functional knee phenotype in the contralateral knee. Journal of Personalized Medicine, 14, 193. Available from: 10.3390/jpm14020193 38392626 PMC10890178

[93] Schweizer, A. , Fürnstahl, P. , Harders, M. , Székely, G. & Nagy, L. (2010) Complex radius shaft malunion: osteotomy with computer‐assisted planning. Hand (New York, NY), 5, 171–178. Available from: 10.1007/S11552-009-9233-4 PMC2880679 19826878

[94] Siboni, R. , Vialla, T. , Joseph, E. , LiArno, S. , Faizan, A. , Martz, P. et al. (2022) Coronal and sagittal alignment of the lower limb in Caucasians: analysis of a 3D CT database. Orthopaedics & Traumatology: Surgery & Research, 108, 103251. Available from: 10.1016/j.otsr.2022.103251 35183757

[95] Subburaj, K. , Ravi, B. & Agarwal, M. (2010) Computer‐aided methods for assessing lower limb deformities in orthopaedic surgery planning. Computerized Medical Imaging and Graphics, 34, 277–288. Available from: 10.1016/J.COMPMEDIMAG.2009.11.003 19963346

[96] Tanoğlu, O. , Subaşı, İ.Ö. , Gökgöz, M.B. & Arıcan, G. (2021) Is proximal tibia sufficient for accurate measurement of tibial slope angles on three‐dimensional tomography‐based anatomical models? Current Medical Imaging, 17, 1419–1424. Available from: 10.2174/1573405617666210806150938 34365952

[97] Tarassoli, P. , Warnock, J.M. , Lim, Y.P. , Jagota, I. & Parker, D. (2024) Large multiplanar changes to native alignment have no apparent impact on clinical outcomes following total knee arthroplasty. Knee Surgery, Sports Traumatology, Arthroscopy, 32, 432–444. Available from: 10.1002/ksa.12044 38294963

[98] Teng, Y. , Mizu‐uchi, H. , Xia, Y. , Akasaki, Y. , Akiyama, T. , Kawahara, S. et al. (2021) Axial but not sagittal hinge axis affects posterior tibial slope in medial open‐wedge high tibial osteotomy: a 3‐dimensional surgical simulation study. Arthroscopy: The Journal of Arthroscopic & Related Surgery, 37, 2191–2201. Available from: 10.1016/J.ARTHRO.2021.01.063 33581296

[99] Tiefenboeck, S. , Sesselmann, S. , Taylor, D. , Forst, R. & Seehaus, F. (2022) Preoperative planning of total knee arthroplasty: reliability of axial alignment using a three‐dimensional planning approach. Acta Radiologica, 63, 1051–1061. Available from: 10.1177/02841851211029076 34229468

[100] Twiggs, J.G. , Dickison, D.M. , Kolos, E.C. , Wilcox, C.E. , Roe, J.P. , Fritsch, B.A. et al. (2018) Patient variation limits use of fixed references for femoral rotation component alignment in total knee arthroplasty. The Journal of Arthroplasty, 33, 67–74. Available from: 10.1016/j.arth.2017.08.023 28927560

[101] Van Genechten, W. , Van Haver, A. , Bartholomeeusen, S. , Claes, T. , Van Beek, N. , Michielsen, J. et al. (2023) Impacted bone allograft personalised by a novel 3D printed customization kit produces high surgical accuracy in medial opening wedge high tibial osteotomy: a pilot study. Journal of Experimental Orthopaedics, 10, 24. Available from: 10.1186/s40634-023-00593-0 36917322 PMC10012299

[102] Vanhove, F. , Noppe, N. , Fragomen, A.T. , Hoekstra, H. , Vanderschueren, G. & Metsemakers, W.J. (2019) Standardization of torsional CT measurements of the lower limbs with threshold values for corrective osteotomy. Archives of Orthopaedic and Trauma Surgery, 139, 795–805. Available from: 10.1007/S00402-019-03139-1 30737593

[103] Veerman, Q.W.T. , Ten Heggeler, R.M. , Tuijthof, G.J.M. , de Graaff, F. , Fluit, R. & Hoogeslag, R.A.G. (2024) Three‐dimensional hinge axis orientation contributes to simultaneous alignment correction in all three anatomical planes in opening‐wedge high tibial osteotomy. Arthroscopy, Sports Medicine, and Rehabilitation, 6, 100888. Available from: 10.1016/j.asmr.2024.100888 38356465 PMC10864846

[104] Victor, J. , Van Doninck, D. , Labey, L. , Innocenti, B. , Parizel, P.M. & Bellemans, J. (2009) How precise can bony landmarks be determined on a CT scan of the knee? The Knee, 16, 358–365. Available from: 10.1016/J.KNEE.2009.01.001 19195896

[105] Vuurberg, G. , Dahmen, J. , Dobbe, I.G.G. , Kleipool, R.P. , Hayat, B. , Sierevelt, I.N. et al. (2022) Lower leg symmetry: a Q3D‐CT analysis. Surgical and Radiologic Anatomy, 44, 851–860. Available from: 10.1007/s00276-022-02940-9 35534775 PMC9246803

[106] Wai Hung, C.L. , Wai Pan, Y. , Kwong Yuen, C. , Hon Bong, L. , Lei Sha, L.W. & Ho Man, S.W. (2009) Interobserver and intraobserver error in distal femur transepicondylar axis measurement with computed tomography. The Journal of Arthroplasty, 24, 96–100. Available from: 10.1016/J.ARTH.2007.11.014 18534429

[107] Wakelin, E.A. , Tran, L. , Twiggs, J.G. , Theodore, W. , Roe, J.P. , Solomon, M.I. et al. (2018) Accurate determination of post‐operative 3D component positioning in total knee arthroplasty: the AURORA protocol. Journal of Orthopaedic Surgery and Research, 13, 275. Available from: 10.1186/s13018-018-0957-0 30376865 PMC6208069

[108] Wu, G. , Siegler, S. , Allard, P. , Kirtley, C. , Leardini, A. , Rosenbaum, D. et al. (2002) ISB recommendation on definitions of joint coordinate system of various joints for the reporting of human joint motion—part I: ankle, hip, and spine. Journal of Biomechanics, 35, 543–548. Available from: 10.1016/S0021-9290(01)00222-6 11934426

[109] Xing, Q. , Han, R. , Li, Y. , Yang, W. & Chen, J.X. (2013) Automatically assessing limb alignment and hip fracture using 3D models. Computing in Science & Engineering, 15, 10–20. Available from: 10.1109/MCSE.2012.107

[110] Yamagami, R. , Inui, H. , Taketomi, S. , Kono, K. , Kawaguchi, K. , Sameshima, S. et al. (2022) Proximal tibial morphology is associated with risk of trauma to the posteromedial structures during tibial bone resection reproducing the anatomical posterior tibial slope in bicruciate‐retaining total knee arthroplasty. The Knee, 36, 1–8. Available from: 10.1016/j.knee.2022.03.008 35381571

[111] Yang, G. , Wang, Z. , Wen, X. , Jiang, Z. , Qi, X. & Yang, C. (2016) The relationship between the midpoints connecting the tibial attachments of the anterior and posterior cruciate ligaments and the transepicondylar axis: in vivo three‐dimensional measurement in the Chinese population. The Knee, 23, 777–784. Available from: 10.1016/J.KNEE.2016.05.003 27329992

[112] Yang, Y. , Zeng, X. , Jin, Y. , Zhu, Z. , Tsai, T.‐Y. , Chen, J. et al. (2022) The presence of cartilage affects femoral rotational alignment in total knee arthroplasty. Frontiers in Surgery, 9, 802631. Available from: 10.3389/fsurg.2022.802631 35252329 PMC8888858

[113] Yue, B. , Varadarajan, K.M. , Ai, S. , Tang, T. , Rubash, H.E. & Li, G. (2011) Differences of knee anthropometry between Chinese and white men and women. The Journal of Arthroplasty, 26, 124–130. Available from: 10.1016/J.ARTH.2009.11.020 PMC3740371 20149574

[114] Zhang, L.S. , Zhou, H. , Zhang, J.C. , Zhang, Q. , Chen, X.Y. & Feng, S. (2022) Different tibial rotational axes can be applied in combination according to the tibial tuberosity‐posterior cruciate ligament distance in total knee arthroplasty. BMC Musculoskeletal Disorders, 23, 906. Available from: 10.1186/s12891-022-05859-9 36217137 PMC9549616

[115] Zhang, R.‐Y. , Su, X.‐Y. , Zhao, J.‐X. , Li, J.‐T. , Zhang, L.‐C. & Tang, P.‐F. (2020) Three‐dimensional morphological analysis of the femoral neck torsion angle—an anatomical study. Journal of Orthopaedic Surgery and Research, 15, 192192. Available from: 10.1186/s13018-020-01712-8 PMC7251911 32460899

[116] Zhang, Y. , Wang, J. , Xiao, J. , Zhao, L. , Li, Z. , Yan, G. et al. (2014) Measurement and comparison of tibial posterior slope angle in different methods based on three‐dimensional reconstruction. The Knee, 21, 694–698. Available from: 10.1016/J.KNEE.2014.01.008 24565940

