# Supplementary material for: High variability exists in 3D leg alignment analysis, but underlying principles that might lead to agreement on a universal framework could be identified: A systematic review
Source: Knee Surg Sports Traumatol Arthrosc. 2024 Oct 26;33(6):2063–77. doi: 10.1002/ksa.12512 (PMC12104779; doi:10.1002/ksa.12512)
Supplement: Supplementary file 1 — Supporting information. [file KSA-33-2063-s001.pdf]

SUPPLEMENTARY INFORMATION

Supplementary information to:

Title High Variability Exists In 3D Leg Alignment Analysis, But Underlying Principles That Might Lead To Agreement On A Universal Framework Could Be Identified: A Systematic Review

Journal KSSTA

Authors Quinten W.T. Veerman, Romy M. ten Heggeler, Gabriëlle J.M. Tuijthof, Feike de Graaff, René Fluit, Roy A.G. Hoogeslag

Corresponding author Quinten W.T. Veerman

Table 1. Search string applied in the databases of Scopus, PubMed and Embase.

Scopus	TITLE-ABS (*3d* OR *3-d* OR *3-dimension** OR *"3 dimension*"* OR *threedimension** OR *three-dimension** OR *"three dimension*"*) AND TITLE-ABS ((*compute** AND *tomography*) OR *ct*) AND TITLE-ABS (*femur* OR *femor** OR *tibia** OR *leg* OR *knee* OR *limb*) AND TITLE-ABS (*alignment* OR *angle* OR *slope* OR *line* OR *point* OR *landmark* OR *axis* OR *axes* OR *plane* OR *coordinate*) AND TITLE-ABS (*measur** OR *calculat** OR *automat** OR *defin** OR *assess** OR *mark**) AND (LIMIT-TO (LANGUAGE , *"English"*) OR LIMIT-TO (LANGUAGE , *"German"*))
PubMed	((3d[Title/Abstract] OR 3-d[Title/Abstract] OR 3-dimension*[Title/Abstract] OR "3 dimension*"[Title/Abstract] OR threedimension*[Title/Abstract] OR three-dimension*[Title/Abstract] OR "three dimension*"[Title/Abstract]) AND ((compute*[Title/Abstract] AND tomography* [Title/Abstract]) OR ct [Title/Abstract]) AND (femur[Title/Abstract] OR femor*[Title/Abstract] OR tibia*[Title/Abstract] OR leg[Title/Abstract] OR knee[Title/Abstract] OR limb[Title/Abstract]) AND (alignment[Title/Abstract] OR angle[Title/Abstract] OR slope[Title/Abstract] OR line[Title/Abstract] OR point[Title/Abstract] OR landmark[Title/Abstract] OR axis[Title/Abstract] OR axes[Title/Abstract] OR plane[Title/Abstract] OR coordinate[Title/Abstract]) AND (measur*[Title/Abstract] OR calculat*[Title/Abstract] OR automat*[Title/Abstract] OR defin*[Title/Abstract] OR assess*[Title/Abstract] OR mark*[Title/Abstract]))
Embase	((3d or 3-d or 3-dimension* or "3 dimension*" or threedimension* or "three-dimension*" or "three dimension*") and ((compute* and tomography) or ct) and (femur or femor* or tibia* or leg or knee or limb) and (alignment or angle or slope or line or point or landmark or axis or axes or plane or coordinate) and (measur* or calculat* or automat* or defin* or assess* or mark*)).ab,ti.

Table 2. Overview of the study and patient characteristics. Arranged by number of subjects included per level of automation.

Study	Year	Subjects, n (out of patients)	Age, y, mean ± SD (Range)	Alignment-parameters on F/T/L*
		**Automated methods**		
Hartel [23]	2016	1070 (1070)	65 (19 – 109)	F
Micicoi [60]	2020	758 (758)	58.5±16.4	F/T/L
Pangaud [67]	2020	756 (378)	58.3 (18 – 92)	T
Siboni [75]	2022	586	61.9±15	F/T
Jacquet [32]	2019	466 (233)	61.2±15.4	F/T/L
León-Muñoz [47]	2021	385 (322)	70.5±8.2	F/T/L
León-Muñoz [46]	2020	227 (183)	NR	F/T/L
Cho [10]	2015	202	f: 54, m: 50	F
Berryman [5]	2014	44	f: 74.4±15.3<br>m: 74.5±7.7	F
Renault [70]	2018	24	NR	NA
Fischer [17]	2020	20	m: 58 (median)<br>f: 41 (median)	F
Vuurberg [85]	2022	20	f: 34.0±10.3<br>m: 37.7±11.1	NA
Miranda [61]	2010	10	58.3±11.1	NA
Kai [38]	2014	5 (5)	65±11.2	NA
Amirtharaj [2]	2018	3 (3)	21, 21, 43	T
Subburaj [76]	2010	1	NR	F/T/L
Modenese [62]	2021	NR	NR	NA
Xing [88]	2013	NR	NR	F/T/L
		**Semi-automated methods**		

Meric [59]	2015	13546	65.4±10.3	F
Tanoglu [77]	2021	400 (200)	47.1±10.4	T
Zhang [94]	2020	213	69.41± 9.21	F
Kawahara [39]	2022	115 (98)	Varus(43): 73.7 ±8.3, Valgus(40): 73.1±8.6, Healthy(32): 31.3±3.9	F/T
Hoch [26]	2022	100 (50)	NR	F
Jud [34]	2020	85	40.4 (15-64)	L
Okamoto [66]	2014	75 (65)	77 (57 – 89)	F
Li [49]	2022	74 (46)	65.7±9.25	F
Jung [37]	2024	70 (63)	57.6±6.1	T
Sasaki [73]	2023	66 (38)	70.2±8.5	T
Brunner [6]	2023	60	(18-50)	F/T
Roth [71]	2021	59 (55)	43.5±8.4	L
Hoch [27]	2020	54 (51)	46 (22 – 67)	T
Chalmers [7]	2023	50	NR	F
Chalmers [8]	2021	40 (36)	63±7.1 (45 – 74)	T
Chung [11]	2022	30	56.2±2.7	T
Preston [68]	2022	28	NR	F
Jud [35]	2020	7	21.9±5.9	F/T
Victor [84]	2009	6	(78 – 87)	F/T
Hancock [22]	2013	5	(61 – 87)	F/T
			**Manual methods**	
Huan [30]	2022	879	74±7	L
Twiggs [81]	2018	726	69.1±8.6	F
Liu [53]	2022	444 (444)	66.4±9.3	F/T
Liu [55]	2022	443 (443)	66.5±9.3	F
Liu [54]	2024	434 (434)	66.4±9.3	F/T/L
Liu [56]	2021	381 (381)	66.5±8.9	F
Tarassoli [78]	2024	296 (261)	70.3 (51-91)	F/T
Meier [58]	2020	234 (234)	62.9±8.72	T
Lei [45]	2020	196	NR	F/T
Vanhove [83]	2019	88 F, 94 T	64 (50 – 85)	L
Zhang [95]	2014	160 (80)	31.38 (20 – 45)	T
Liu [52]	2023	150 (75)	70.05±12.34	F
Sato [74]	2022	140 (73)	76.1 (59 – 91)	F
Zhang [93]	2022	135	65.8±7.7 (43 – 89)	T
Liu [51]	2023	120	50.18±10.92 (26-77)	F
Ikuta [31]	2020	106 (81)	48.4±19.9	NA
Ho [25]	2017	100 (100)	54 (18 – 81)	T
Ho [24]	2021	100 (100)	54 (18 – 81)	T
Teng [79]	2021	93(93)	75.1±8.1	T
Ma [57]	2017	87	(18 – 92)	F/T
Yang [91]	2022	83	36.2±14.3	F
Li [50]	2021	78 (78)	NR	NA
Flury [18]	2022	77 (60)	21.8±6.1	F/T
Yue [92]	2011	76(76)	(19.7 – 54)	F/T
An [3]	2024	72	65±5.8 (54-84)	F/T
Qin [69]	2018	64(64)	(51 – 70)	NA
Tiefenboeck [80]	2021	61(51)	NR	F/T
Degen [13]	2020	60(30)	f: 35.8±11.4<br>m: 32.2±10.6	F/T/L
Lei [44]	2022	60 (59)	67.7±8.2	F
Ohmori [65]	2020	55	Varus(24): 73.9±7.2, Healthy(31): 61.0±10.5	F
Roth [72]	2023	53	NR	L
Arn Roth [4]	2024	52	NR	T
Kuiper [42]	2023	50	f: 51 (34 – 76)<br>m: 61 (30 – 76)	F/T
León-Muñoz [48]	2021	50	71.34±5.65	F
Yamagami [89]	2022	50	72.9 (56 – 85)	T
Hodel **[28]**	2024	43 (41)	43.6±10.1	L
Kim [40]	2020	42(41)	56.7 (44 – 73)	T
Yang [90]	2016	32(32)	27.8±3.5	T
Enomoto [15]	2013	32	71.3±7.8	T
Cho [9]	2022	30	>19	F/T
Van Genechten [82]	2023	30	48±13	F/T
Nedopil [64]	2023	26 (13)	81±10.3	F
Hanada [21]	2020	24(20)	67.9 (54 – 89)	F/T
Eckhoff [14]	2007	23	(48 – 91)	F
Lee [43]	2017	23(21)	45 (19 – 59)	T
Moon [63]	2015	19(17)	45.9 (19 – 66)	T
Hodel [29]	2024	19(18)	29±5 (21 – 41)	L
Factor [16]	2024	18	71 ± 8.8	F/T
Jörgens [33]	2022	13	NR	T
Wai Hung [86]	2009	10	NR	F
Citak [12]	2010	8	NR	F

Jud [36]	2020	7	NR	L
Fürmetz [19]	2018	3(3)	(16 – 75)	F/T/L
Wakelin [87]	2018	2	NR	F/T
Adachi [1]	2023	1	NA	F/T

Abbreviations: F , femur; f, female; m, male; NA, not applicable (i.e., no leg alignment parameters obtained); NR, not reported; L, leg; T, tibia.

*L = alignment parameters of the leg (i.e., hip knee ankle angle; joint line convergence angle; mechanical axis deviation).

Table 3. Level of Evidence (LOE) of included studies, arranged in order of level of quality assessment per LOE[a]

		Critical Appraisal[b]															
Study	Design	1	2	3	4	5	6	7	8	9	10	11	12	13	14	T/A[c]	%
								**LOE III**									
Zhang [94]	Retrospective cohort	y	y	y	y	y	CD	NA	NA	NA	NA	y	NA	y	y	8/9	89
Vanhove [83]	Retrospective cohort	y	y	y	y	y	CD	NA	NA	NA	NA	y	NA	y	y	8/9	89
Flury [18]	Retrospective cohort	y	y	NA	y	y	n	NA	NA	NA	NA	y	NA	y	y	7/8	88
Liu [56]	Retrospective cohort	y	y	y	y	n	y	NA	NA	y	n	y	y	y	y	10/12	83
Liu [55]	Retrospective cohort	y	y	y	y	n	y	y	y	y	n	y	n	y	y	11/14	79
León-Muñoz [47]	Prospective cohort	y	y	NR	y	y	CD	NA	NA	NA	NA	y	NA	y	y	7/9	78
Li [49]	Prospective cohort	y	y	y	y	n	n	NA	NA	NA	NA	y	NA	y	y	7/9	78
Liu [53]	Retrospective cohort	y	y	y	y	n	n	NA	NA	NA	NA	y	NA	y	y	7/9	78
Tanoglu [77]	Retrospective cohort	y	y	NR	y	y	n	NA	NA	NA	NA	y	NA	y	y	7/9	78
Yang [91]	Retrospective cohort	y	y	NR	y	y	n	NA	NA	NA	NA	y	NA	y	y	7/9	78
Huan [30]	Retrospective cohort	y	y	y	y	y	n	NA	NA	NA	NA	y	NA	y	n	7/9	78
León-Muñoz [46]	Prospective cohort	y	y	NR	y	y	y	y	NA	y	n	y	n	y	y	10/13	77
Siboni [75]	Retrospective cohort	y	y	NA	y	n	n	NA	NA	NA	NA	y	NA	y	y	6/8	75
Zhang [93]	Prospective cohort	y	y	y	y	y	y	y	y	n	n	y	n	y	n	10/14	71
Hodel [28]	Retrospective cohort	y	y	y	y	n	y	y	y	n	n	y	n	y	y	10/14	71
Kim [40]	Retrospective cohort	y	n	NR	CD	y	y	y	NA	y	y	y	n	y	y	9/13	69
Liu [54]	Retrospective cohort	y	y	y	y	n	n	NA	NA	NA	NA	y	NA	y	n	6/9	67
Jung [37]	Retrospective cohort	y	y	NR	y	n	n	NA	NA	NA	NA	y	NA	y	y	6/9	67
Liu [51]	Retrospective cohort	y	y	y	y	n	n	NA	NA	NA	NA	y	NA	y	n	6/9	67
Hartel [23]	Retrospective cohort	y	y	NR	y	n	CD	NA	NA	NA	NA	y	NA	y	y	6/9	67
Li [50]	Prospective cohort	y	y	NR	y	n	CD	NA	NA	NA	NA	y	NA	y	y	6/9	67
Pangaud [67]	Retrospective cohort	y	y	n	n	y	CD	NA	NA	NA	NA	y	NA	y	y	6/9	67
Sato [74]	Prospective cohort	y	y	NR	y	n	n	NA	NA	NA	NA	y	NA	y	y	6/9	67
Twiggs [81]	Prospective cohort	y	y	NR	y	n	n	NA	NA	NA	NA	y	NA	y	y	6/9	67
Liu [52]	Retrospective cohort	y	y	NR	y	y	y	y	n	y	n	y	n	y	n	9/14	64
Cho [10]	Human cadaver	y	y	NA	y	n	y	NA	NA	n	n	y	n	y	y	7/11	64
Hoch [27]	Retrospective cohort	y	y	y	y	n	y	y	n	y	n	y	n	y	n	9/14	64
Jud [34]	Retrospective cohort	y	y	y	y	n	y	y	n	y	n	y	n	y	n	9/14	64
Sasaki [73]	Prospective cohort	y	y	NR	y	n	y	y	y	y	n	y	n	y	n	9/14	64
Van Genechten [82]	Prospective cohort	y	y	y	y	n	y	y	y	n	n	y	n	y	n	9/14	64
Brunner [6]	Retrospective cohort	y	y	NA	NR	n	y	y	y	y	n	y	n	y	n	8/13	62
Okamoto [66]	Prospective cohort	y	n	NR	NR	y	y	NA	y	y	n	y	y	y	n	8/13	62
Yamagami [89]	Prospective cohort	y	y	y	y	y	y	NA	n	n	n	y	n	y	n	8/13	62
Jacquet [32]	Retrospective cohort	y	y	n	n	y	y	NA	NA	n	n	y	n	y	y	7/12	58
Micicoi [60]	Retrospective cohort	y	y	n	n	y	y	NA	NA	n	n	y	n	y	y	7/12	58
Roth [71]	Retrospective cohort	y	y	NR	y	n	y	NA	NA	n	n	y	n	y	y	7/12	58
Teng [79]	Retrospective cohort	y	n	NR	y	y	y	NA	NA	y	n	y	n	y	n	7/12	58
Hodel [29]	Retrospective cohort	y	y	NR	y	n	y	y	y	n	n	y	n	y	n	8/14	57
Tarassoli [78]	Retrospective cohort	y	y	NR	y	n	y	y	n	y	n	y	n	y	n	8/14	57
Roth [72]	Retrospective cohort	y	y	NR	y	n	n	NA	NA	NA	NA	y	NA	y	n	5/9	56
Cho [9]	Retrospective cohort	y	n	y	y	n	n	NA	NA	NA	NA	y	NA	y	n	5/9	56
Fischer [17]	Human cadaver	y	y	NR	n	y	n	NA	NA	NA	NA	y	NA	y	n	5/9	56
Hanada [21]	Retrospective cohort	y	y	NR	y	n	n	NA	NA	NA	NA	y	NA	y	n	5/9	56
Ho [25]	Retrospective cohort	y	n	NR	y	n	n	NA	NA	NA	NA	y	NA	y	y	5/9	56
Ho [24]	Retrospective cohort	y	n	NR	y	n	n	NA	NA	NA	NA	y	NA	y	y	5/9	56

Ikuta [31]	Prospective cohort	y	n	NR	y	n	n	NA	NA	NA	NA	y	NA	y	y	5/9	56
Lei [44]	Retrospective cohort	y	y	NR	y	n	n	NA	NA	NA	NA	y	NA	y	n	5/9	56
Lei [45]	Retrospective cohort	y	y	NR	NR	n	n	NA	NA	NA	NA	y	NA	y	y	5/9	56
Lee [43]	Retrospective cohort	y	n	y	CD	y	CD	NA	NA	NA	NA	y	NA	y	n	5/9	56
León-Muñoz [48]	Prospective cohort	y	n	NR	NR	y	n	NA	NA	NA	NA	y	NA	y	y	5/9	56
Meier [58]	Retrospective cohort	y	n	NR	CD	y	n	NA	NA	NA	NA	y	NA	y	y	5/9	56
Tiefenboeck [80]	Retrospective cohort	y	y	NR	y	n	n	NA	NA	NA	NA	y	NA	y	n	5/9	56
Degen [13]	Human cadaver	y	n	NA	NR	y	y	NA	NA	n	n	y	n	y	y	6/11	55
Ohmori [65]	Case-control	y	y	NR	y	n	y	NA	n	n	n	y	n	y	y	7/13	54
Chung [11]	Retrospective cohort	y	y	NR	y	n	y	NA	n	n	n	y	n	y	n	6/13	46
Kawahara [39]	Prospective cohort	y	n	NR	NR	n	y	NA	y	n	n	y	n	y	y	6/13	46
Enomoto [15]	Prospective cohort	y	y	NR	NR	n	n	NA	NA	NA	NA	y	NA	y	n	4/9	44
Ma [57]	Human cadaver & retrospective cohort	y	y	NR	NR	n	n	NA	NA	NA	NA	y	NA	y	n	4/9	44
Yue [92]	Prospective cohort	y	n	NR	n	n	n	NA	NA	NA	NA	y	NA	y	y	4/9	44
Chalmers [8]	Retrospective cohort	y	y	NR	NR	n	n	NA	NA	NA	NA	y	NA	y	n	4/9	44
An [3]	Retrospective cohort	y	y	NR	NR	n	n	NA	NA	NA	NA	y	NA	y	n	4/9	44
Hoch [26]	Human cadaver	y	n	NA	y	n	n	NA	NA	NA	NA	y	NA	NA	n	3/7	43
Yang [90]	Prospective cohort	y	n	NR	NR	n	y	NA	NA	n	n	y	n	y	y	5/12	42
Zhang [95]	Prospective cohort	y	n	NR	NR	n	y	NA	NA	n	n	y	n	y	y	5/12	42
Meric [59]	Retrospective cohort	y	n	NR	NR	n	y	NA	n	n	n	y	y	y	n	5/13	38
Berryman [5]	Human cadaver	y	n	NA	NR	n	n	NA	NA	NA	NA	y	NA	y	n	3/8	38
Eckhoff [14]	Human cadaver	y	n	NA	NR	n	n	NA	NA	NA	NA	y	NA	y	n	3/8	38
Jörgens [33]	Human cadaver	y	n	NA	NR	n	n	NA	NA	NA	NA	y	NA	y	n	3/8	38
Kuiper [42]	Retrospective cohort	y	y	NR	y	n	n	NA	NA	NA	NA	y	NA	y	n	3/9	33
Qin [69]	Retrospective cohort	y	n	NR	NR	n	n	NA	NA	NA	NA	y	NA	y	n	3/9	33
Vuurberg [85]	Prospective cohort	y	n	NR	NR	n	n	NA	NA	NA	NA	y	NA	y	n	3/9	33
Preston [68]	Prospective cohort	y	n	n	NR	n	n	NA	NA	NA	NA	y	NA	y	n	3/9	33
Factor [16]	Retrospective cohort	y	y	NR	NR	n	n	NA	NA	NA	NA	n	NA	y	n	3/9	33
Chalmers [7]	Human cadaver	y	n	NA	NR	n	n	NA	NA	NA	NA	y	NA	NA	n	2/7	29
Arn Roth [4]	Retrospective cohort	n	n	NA	NA	n	n	NA	NA	NA	NA	y	NA	NA	n	1/6	17
**LOE IV**																	
Jud [35]	Prospective cohort	y	y	y	y	n	y	y	y	n	n	y	n	y	n	9/14	64
Hancock [22]	Human cadaver	y	y	NA	y	n	y	NA	NA	n	n	y	n	y	n	6/11	55
Nedopil [64]	Human cadaver	y	y	NA	NR	n	n	NA	NA	NA	NA	y	NA	y	n	4/8	50
Adachi [1]	Model	y	y	NA	NA	n	n	NA	NA	NA	NA	y	NA	NA	n	3/6	50
Citak [12]	Human cadaver	y	n	NA	NR	n	y	NA	NA	y	n	y	n	y	n	5/11	45
Fürmetz [19]	Human cadaver	y	n	NA	y	n	y	NA	NA	n	n	y	n	y	n	5/11	45
Moon [63]	Prospective cohort	y	n	y	CD	n	n	NA	NA	NA	NA	y	NA	y	n	4/9	44
Jud [36]	Retrospective cohort	y	n	NR	NR	n	y	NA	y	n	n	y	n	y	n	5/13	38
Amirtharaj [2]	Human cadaver	y	n	NA	n	n	CD	NA	NA	NA	NA	y	NA	y	n	3/8	38
Wakelin [87]	Retrospective cohort	y	y	NR	NR	n	n	NA	NA	NA	NA	y	NA	NA	n	3/8	38
Victor [84]	Human cadaver	y	n	NA	NR	n	n	NA	NA	NA	NA	y	NA	y	n	3/8	38
Wai Hung [86]	Human cadaver	y	n	NA	NR	n	n	NA	NA	NA	NA	y	NA	y	n	3/8	38
Miranda [61]	Human cadaver	y	n	NA	NR	n	n	NA	NA	NA	NA	y	NA	y	n	3/8	38
Kai [38]	Prospective cohort	y	n	NR	NR	n	n	NA	NA	NA	NA	y	NA	y	n	3/9	33
Renault [70]	Retrospective cohort	y	n	NR	NR	n	n	NA	NA	NA	NA	y	NA	y	n	3/9	33
Subburaj [76]	Case	y	n	NR	n	n	n	NA	NA	NA	NA	y	NA	y	n	3/9	33
Xing [88]	Case	y	n	NR	n	n	n	NA	NA	NA	NA	y	NA	y	n	3/9	33
Modenese [62]	Case-control	y	n	NR	NR	n	n	NA	NA	NA	NA	y	NA	NR	n	2/9	22

[a]Level of evidence (LOE) according to the Oxford Center of Evidence-Based Medicine criteria and critical appraisal according to the Quality Assessment Tool for Observational Cohort and Cross-Sectional Studies from the National Institutes of Health. [b]Critical appraisal items were rated as yes, no, cannot determine (e.g., not completely reported; CD), not applicable (NA) or not reported (NR): 1, clearly stated research question or objective; 2, clearly specified or defined study population; 3, participation rate of eligible persons => 50%; 4, subjects selected from same or similar populations + in- and exclusion criteria prespecified and uniformly applied; 5, sample size justification, power description, or variance and effect estimates provided; 6, exposure of interest measured prior to the outcome being measured; 7, timeframe sufficient to reasonably expect to see an association between exposure and outcome; 8, for exposures that can vary in level, the study examined different levels of exposure related to the outcome; 9, exposure measures clearly defined, valid, reliable, and implemented consistently across all study participants; 10, exposure assessed more than once over time;

11, outcome measures were clearly defined, valid, reliable, and consistently applied across all study participants; 12, outcome assessors were blinded to the exposure status of participants; 13, loss to follow-up after baseline =< 20%; 14, key potential confounding variables measured and adjusted statistically for their impact on the relationship between exposure and outcome. [c]T = sum of CA items scored / A = sum of applicable CA items.

Abbreviations: % = T/A * 100%, CD = cannot determine (e.g., not completely reported), LOE = level of evidence, n = no, NA = not applicable, NR = not reported, y = yes.

Table 4. Overview of methods to derive femoral axes and joint orientations from 3D bone models to perform 3D knee-related alignment analysis. For the femur, these methods were grouped into three categories: landmark method (a line between two landmark points), calculated line method (a line calculated from a multitude of points), and best fit geometrical shape method (a geometrical shape with best fit to a multitude of points on the round articular surface from which a central axis can be extracted). For each category, the explicit method per study is reported, with the landmark method described by a landmark pair followed by reported methods for each of the landmarks.

Axis or joint orientation	Category	Method		
mFA	Landmark	Landmark pair<br>FHC – KJC [21, 29, 32, 36, 59, 60, 67, 75]	Proximal<br>Manual<br>• Based on 4 reference points [21]<br>• Explicit method unknown [29, 36]<br>Semi-automatic: spherical center fitted to FH<br>• Explicit method unknown [59]<br>Automatic<br>• Explicit method unknown, with algorithm-based measurements on corresponding bone, and mapped to chosen bone [32, 60, 67]<br>• Explicit method unknown [75]	Distal<br>Manual<br>• Explicit method unknown [21, 29, 36, 59]<br>Automatic<br>• Explicit method unknown, with algorithm-based measurements on corresponding bone, and mapped to chosen bone [32, 60, 67]<br>• Explicit method unknown [75]
		FHC – intercondylar eminence center [4, 18, 22, 28, 34, 42, 71, 72]	Semi-automatic: spherical center fitted to FH<br>• Selected points on FH, followed by sphere fitting FH using LSR [34, 71, 72]<br>• Explicit method unknown [4, 18, 22, 28, 42]	Manual<br>• Point equidistant between intercondylar tubercles [22]<br>• Midpoint between midpoint of medial tibial spine and midpoint of lateral tibial spine [42]<br>• Explicit method unknown [4, 18, 28, 34, 71, 72]
		FHC – center intercondylar FN [6, 17, 46, 47, 54, 81]	Manual<br>• Explicit method unknown [6, 54, 81]<br>Automatic<br>• Spherical center FH, explicit method unknown, with use of mean determinations of reference points [46, 47]<br>• Least-squares fitting of a sphere to the vertices of the head mapped from a registered template femur [17]	Manual<br>• Explicit method unknown [6, 54, 81]<br>Automatic<br>• Explicit method unknown, with use of mean determinations of reference points [46, 47]<br>• Acquired based on curvature and local maxima in all three dimensions of the detected boundary between trochlear surface and intercondylar fossa [17]
		FHC – midpoint FEA [49, 52, 57, 66, 82, 91]	Manual<br>• Explicit method unknown [49, 91]<br>Semi-automatic: spherical center fitted to FH<br>• Explicit method unknown [52, 57, 66, 82]	Manual<br>• Midpoint most prominent points medial & lateral epicondyle [49, 52, 57]<br>• Explicit method unknown [66, 82, 91]
		FHC – most cranial point FN [13, 19]	Manual<br>• Midpoint of line in medial view, by connecting midpoint FH posterior and anterior view [19]<br>• Explicit method unknown [13]	Manual<br>• Placed and confirmed in posterior view [19]<br>• Explicit method unknown [13]
		FHC – apex intercondylar FN [10, 44, 45, 53, 55, 56]	Manual<br>• Explicit method unknown [55, 56]<br>Semi-automatic: spherical center fitted to FH<br>• Explicit method unknown [44, 45, 53]<br>Automatic<br>• Explicit method unknown [10]	Manual<br>• Explicit method unknown [44, 45, 53, 55, 56]<br>Automatic<br>• Explicit method unknown [10]
		FHC – midpoint distal femoral joint line [42]	Semi-automatic: spherical center fitted to FH<br>• Explicit method unknown [42]	Manual<br>• Explicit method unknown [42]

		FHC – most anterior point middle FN [84]	Semi-automatic: spherical center fitted to FH<br>• Explicit method unknown [84]	Manual<br>• Explicit method unknown [84]
		FHC – center of the trochlea [9]	Manual<br>• Explicit method unknown [9]	Manual<br>• Explicit method unknown [9]
		FHC – distal point trochlea [3, 7, 74]	Semi-automatic: center best-fit sphere FH<br>• Explicit method unknown [7]<br>Semi-automatic<br>• Point with smallest standard deviation to all points of FH point cloud, iteratively acquired [74]<br>Manual<br>• Explicit method unknown [3]	Manual<br>• Lowest point trochlear groove in coronal and sagittal views [7]<br>• Most distal point trochlear groove [3]<br>• Explicit method unknown [74]
		FHC – FAAD [88]	Semi-automatic: spherical center fitted to FH<br>• 4 selected points on FH, automatic sphere fit [88]	Automatic<br>• Intersection points of fitted line and bone model, with fitted line obtained by skeletonization algorithm [88]
		Proximal point FH – narrowest intercondylar region [83]	Manual<br>• Superficial proximal point projecting in center FH sphere [83]	Manual<br>• Explicit method unknown [83]
DFJ	Landmark	Landmark pair<br>FMCD – FLCD [6, 9, 10, 19, 32, 34, 42, 46, 47, 55, 60, 71, 75, 76, 78, 91]	Medial<br>Manual<br>• Placed in posterior view, confirmed in lateral view [19]<br>• Explicit method unknown [6, 9, 42, 78, 91]<br>• Most distal apex [55]<br>Automatic<br>• Mean of three most distal points along longitudinal axis (acquired through PCA) of K-means clustered medial condyle [34, 71]<br>• Explicit method unknown, with use of mean determinations of reference points [46, 47] or algorithm-based measurements on corresponding bone, and mapped to chosen bone [32, 60]<br>• Explicit method unknown [10, 75, 76]	Lateral<br>Manual<br>• Placed in posterior view, confirmed in lateral view [19]<br>• Explicit method unknown [6, 9, 42, 78, 91]<br>• Most distal apex [55]<br>Automatic<br>• Mean of three most distal points along longitudinal axis (acquired through PCA) of K-means clustered lateral condyle [34, 71]<br>• Explicit method unknown, with use of mean determinations of reference points [46, 47] or algorithm-based measurements on corresponding bone, and mapped to chosen bone [32, 60]<br>• Explicit method unknown [10, 75, 76]
PFCA	Landmark	Landmark pair<br>FMCP – FLCP [5, 9, 10, 12, 16, 17, 19, 23, 26, 35, 47, 48, 50, 51, 56, 57, 59, 78, 80, 81, 83, 84, 86, 91, 93]	Medial<br>Manual<br>• Most posterior apex medial condyle [56], tangent to femur [12, 55, 56, 74, 93]<br>• Placed in caudal view, confirmed in medial view [19]<br>• At largest A-P width in medial view [83]<br>• "Lowest" point in AP-direction of medial posterior condyle [51, 52]<br>• Relative to mFA [84]<br>• Rotation and expansion of (PCA-constructed [35])oriented bounding box of the femur until most posterior point on medial condyle was located [26, 35]<br>• Explicit method unknown [9, 16, 42, 44, 48, 50, 57, 59, 78, 80, 81, 86, 91]<br>Automatic<br>• Proximal using mean determinations of reference points, tangent to femur [47]<br>• Posterior apex [5], tabletop method [5, 23]<br>• Explicit method unknown [10]	Lateral<br>Manual<br>• Most posterior apex lateral condyle [56], tangent to femur [12, 55, 56, 74, 93]<br>• Placed in caudal view, confirmed in lateral view [19]<br>• At largest A-P width in lateral view [83]<br>• "Lowest" point in AP-direction of lateral posterior condyle [51, 52]<br>• Relative to mFA [84]<br>• Rotation and expansion of (PCA-constructed [35]) oriented bounding box of the femur until most posterior point on lateral condyle was located [26, 35]<br>• Explicit method unknown [9, 16, 42, 44, 48, 50, 57, 59, 78, 80, 81, 86, 91]<br>Automatic<br>• Proximal using mean determinations of reference points, tangent to femur [47]<br>• Posterior apex [5], tabletop method [5, 23]<br>• Explicit method unknown [10]

			• Tabletop plane defined by initial most posterior points points of femoral condyles and trochanteric crest, iteratively rotated towards CS defined by tabletop plane, and repeated detection of most posterior points until convergence to unit rotation matrix [17]	• Tabletop plane defined by initial most posterior points points of femoral condyles and trochanteric crest, iteratively rotated towards CS defined by tabletop plane, and repeated detection of most posterior points until convergence to unit rotation matrix [17]
FEA	Landmark	Landmark pair<br>FME – FLE [14, 16, 17, 22, 47, 49, 51-53, 57, 66, 76, 80, 82, 84]	Medial<br>Manual<br>• Most prominent point [22, 49, 51-53, 57, 80]<br>• Most anterior distal prominence over medial aspect of medial condyle [84]<br>• The spot of bone visible on medial condyle, by extension cylinders of FCA [14]<br>• Anterior to medial sulcus [66]<br>• Explicit method unknown [16, 82]<br>Automatic<br>• Explicit method unknown, with use of mean determinations of reference points [47]<br>• Most medial point of distal femur in a unified sagittal plane coordinate system based on the posterior foci of ellipses fitted to the articulating part of the sagittal contours of the femoral condyles, until the dispersion of the posterior foci converges to a minimum, or mapped based on template in the case of large osteophytes at medial rim of articular surface [17]<br>• Explicit method unknown [76]	Lateral<br>Manual<br>• Most prominent point [22, 49, 51-53, 57, 66, 80]<br>• Most anterior distal prominence over lateral aspect of lateral condyle [84]<br>• The spot of bone visible on lateral condyle, by extension cylinders of FCA [14]<br>• Explicit method unknown [16, 82]<br>Automatic<br>• Explicit method unknown, with use of mean determinations of reference points [47]<br>• Most lateral point of distal femur in unified sagittal plane coordinate system based on the posterior foci of ellipses fitted to the articulating part of the sagittal contours of the femoral condyles, until the dispersion of the posterior foci converges to a minimum, or mapped based on template in the case of large osteophytes at lateral rim of articular surface [17]<br>• Explicit method unknown [76]
		Most concave medial point – FLE [3, 45, 51, 52, 54, 56, 59, 65, 78, 80, 81, 84, 86, 87, 91, 93]	Manual<br>• Explicit method unknown [3, 45, 51, 52, 54, 56, 59, 65, 78, 80, 81, 84, 86, 87, 91, 93]	Manual<br>• Most prominent point [45, 51, 52, 54, 56, 59, 78, 80, 86, 91]<br>• Most anterior distal [84]<br>• Lateral apex [3]<br>• Explicit method unknown [65, 81, 87, 93]
FCA	Landmark	Landmark pair<br>Centroid medial condyle – centroid lateral condyle [5, 9]	Medial<br>Manual<br>• Midpoint sphere fitting medial femoral condyle [9]<br>Automatic<br>• Midpoint ellipsoids fitting total medial condyle in coronal and sagittal plane, parallel to PFCA [5]	Lateral<br>Manual<br>• Midpoint sphere fitting lateral femoral condyle [9]<br>Automatic<br>• Midpoint ellipsoids fitting total lateral condyle in coronal and sagittal plane, parallel to PFCA [5]
	Best fit geometrical shape		Manual<br>• Center line cylinders fitting posterior medial and lateral condyle with shared co-axis, with rim of condylar bone left outside cylinder at level of arc traced by the tibia articulating with the femur during knee flexion of 15-115 degrees [14]<br>Semi-automatic<br>• Center line cone fitting selected points posterior condyle, using LSR [22]<br>Semi-automatic<br>• Center line cylinder fitting marked flexion extension surfaces femur [68]	
HJ	Landmark	Landmark pair<br>FHC – TTM [13, 19, 42]	Medial<br>Manual<br>• Midpoint of line in medial view, by connecting midpoint FH posterior and anterior view [19]<br>• Explicit method unknown [13]<br>Semi-automatic: spherical center fitted to FH<br>• Explicit method unknown [42]	Lateral<br>Manual<br>• Placed in posterior view, confirmed in lateral view [19]<br>• Most proximal point greater trochanter [42]<br>• Explicit method unknown [13]

NFA	Landmark	Landmark pair<br>FHC – FNC [7, 12, 13, 19, 23, 26, 42, 47, 56, 74, 94]	Medial<br>Manual<br>• Midpoint of line in medial view, by connecting midpoint FH posterior and anterior view [19]<br>• Explicit method unknown [12, 13, 56]<br>Semi-automatic<br>• Point with smallest standard deviation to all points of FH point cloud, iteratively acquired [74]<br>Semi-automatic: spherical center fitted to FH<br>• Manually selected points, sphere fitted using 'analyze' function 3-Matic [94]<br>• Explicit method unknown [7, 26, 42]<br>Automatic<br>• Spherical center FH, with use of mean determinations of reference points [47]<br>• Explicit method unknown, with algorithm-based measurements on corresponding bone, and mapped to chosen bone [23]	Lateral<br>Manual<br>• Midpoint of line in medial view, by connecting midpoint femoral neck in posterior and anterior view [19]<br>• Explicit method unknown [7, 12, 13, 56]<br>Semi-automatic<br>• Center of mass of resection plane femoral neck, after cutting with a plane normal to a cylinder aligned in the femoral neck and bound to the FHC at 1.25 times the radius of femoral head-fitted sphere [26]<br>• Midpoint of the vertices at the smallest cross-section of femoral neck [42]<br>• Centroid femoral neck, explicit method unknown [74]<br>Automatic<br>• Center of circle obtained by intersection neck base with 2 mm radius increased FH sphere [94]<br>• Explicit method unknown, with use of mean determinations of reference points [47]<br>• Explicit method unknown, with algorithm-based measurements on corresponding bone, and mapped to chosen bone [23]
		Proximal point FH – distal point femoral neck [83]	Manual<br>• Superficial proximal point projecting in center FH sphere [83]	Manual<br>• Superficial distal point projecting in center FH sphere [83]
	Calculated line		Automatic<br>• PCA and LSR of neck surface points at predefined neck range. Range depending on initial FHC and neck base point by minimum derivative. Initial FHC automatically obtained by initial sphere based on Hough transforms FH in 3 planes. Sphere fitted on FH 4-6 mm from initial sphere using least squares fitting (Nelder-Mead) [5]<br>• 3D fitting line through FHC, based on mid-diaphyseal curve. FHC semi-automatically obtained by a spherical center fitted to FH based on 4 selected points and automatic sphere fit [88]<br>• Least-squares fitting of ellipse to neck area mapped from a registered template femur. Refinement by iteratively changing orientation of cutting planes through neck until perimeter of cutting contours converges to a minimum. Neck axis defined by normal of the cutting contour with smallest perimeter [17]<br>• First, centers of medial and lateral neck surfaces were connected. Then, several intersection planes perpendicular to this line were defined, and each intersection with the femoral neck was fit with a best-fit circle. The femoral neck axis was then the best-fit line passing through the centers of the circles using linear regression. [78]	
	Best fit geometrical shape		Semi-automatic<br>• Center line cylinder fitting femoral neck [39]	
aFA	Landmark	Landmark pair<br>FAAP – FAAD [3, 7, 19, 42, 45]	Proximal<br>Manual<br>• Midpoint at 1/3 shaft length [19]. Placed in posterior and anterior view, medial view for midpoint connecting line [19]<br>• Center point femoral medullary cavity 20 cm above knee joint line [45]<br>• Center of canal at cross-section of the femur with the narrowest canal (isthmus) [7]<br>• Medullary cavity midpoint 15cm from knee line [3]<br>Automatic<br>• Midpoint of femoral transverse cross-section 75% of distance along femoral axis [42]	Distal<br>Manual<br>• Midpoint at 2/3 shaft length [19]. Placed in posterior and anterior view, medial view for midpoint connecting line [19]<br>• Center point femoral medullary cavity 10 cm above knee joint line [45]<br>• Lowest point trochlear groove in coronal and sagittal views [7]<br>• Medullary cavity midpoint 5cm from knee line [3]<br>Automatic<br>• Midpoint of femoral transverse cross-section 15% of distance along femoral axis [42]

Calculated line	Automatic<br>• LSR PCA [10, 34, 71], of all femur points [34, 71], or unknown shaft length [10]<br>• Best fit curve, spherical center at 2/8 to 7/8 femur length [76]<br>• Skeletonization algorithm, resulting in curve, with fitted line resulting in FAAP and FAAD intersection points with bone model [88]<br>• Line with equal distance to anterior, posterior, medial and lateral rims of femoral shaft, explicit method unknown [57]<br>• Fitting a line to the center of the femoral shaft, explicit method unknown [16]
Best fit geometrical shape	• Major axis of least-squared-fitted ellipsoid to middle two quarters of length femur [17]

Abbreviations: aFA, anatomical femoral axis; mFA, mechanical femoral axis; DFJ, distal femoral joint orientation; FAAP, proximal femoral anatomical axis; FAAD, distal femoral anatomical axis; FCA, femoral condylar axis; FEA, femoral epicondylar axis; FH, femoral head; FHC, femoral head center; FLCD, distal femoral lateral condyle; FLCP, posterior femoral lateral condyle; FLE, lateral femoral epicondyle; FMCD, distal medial femoral condyle; FMCP, posterior medial femoral condyle; FME, medial femoral epicondyle; FN, femoral notch; FNC, narrowest femoral neck center; HJ, hip joint orientation; KJC, knee joint center; LSR, least square regression; NFA neck femur axis; PCA, principal component analysis; PFCA, posterior femoral condylar axis; SEA, surgical epicondylar axis; TTM, tip trochanter major.

Table 5. Overview of methods to derive tibial axes and joint orientations from 3D bone models to perform 3D knee-related alignment analysis. For the tibia, the methods were grouped into four categories: landmark method (a line between two landmark points), calculated line method (a line calculated from a multitude of points), best fit geometrical shape method (a geometrical shape with best fit to a multitude of points on the round articular surface from which a central axis can be extracted), and calculated plane method (a plane fitting a multitude of points on a flat articular surface from which either the normal or the intersecting line with an anatomical reference plane can be extracted). For each category, the explicit method per study is reported, with the landmark method described by a landmark pair followed by reported methods for each of the landmarks.

Joint orientation line or axis	Category	Method		
mTA	Landmark	Landmark pair<br>Intercondylar eminence center – midpoint tibial plafond [9, 13, 19, 34, 42, 71, 82, 84]	Proximal<br>Manual<br>• Placed in posterior view, confirmed in cranial view [19]<br>• Projected on the bony surface along the tibial shaft axis [84]<br>• Midpoint between midpoint of medial tibial spine and midpoint of lateral tibial spine [42]<br>• Explicit method unknown [9, 13, 34, 71, 82]	Distal<br>Manual<br>• Placed and confirmed in caudal view [19]<br>• Centre best-fit circle tibial plafond [84]<br>• Midpoint between midpoints of medial and lateral plafond of the distal tibial joint [42]<br>• Explicit method unknown [9, 13, 82]<br>Automatic<br>• Centroid articular surface points distal tibia and fibula with specific threshold to talus using CPD [34, 71]
		KJC – AJC [29, 32, 36, 60, 67, 75]	Manual<br>• Explicit method unknown [29, 36]<br>Automatic<br>• Explicit method unknown, with algorithm-based measurements on corresponding bone, and mapped to chosen bone [32, 60, 67]<br>• Explicit method unknown [75]	Manual<br>• Explicit method unknown [29, 36]<br>Automatic<br>• Explicit method unknown, with algorithm-based measurements on corresponding bone, and mapped to chosen bone [32, 60, 67]<br>• Explicit method unknown [75]
		Intercondylar eminence center – TIMA center [3, 22, 46, 47, 57, 76]	Manual<br>• Midpoint peaks of eminences [57]<br>• Explicit method unknown [22]<br>• Midpoint of highest points of medial and lateral tibial intercondylar eminences [3]<br>Automatic<br>• Midpoint of connecting line medial and lateral intercondylar tubercles, with use of mean determinations of reference points [46, 47]<br>• Midpoint of connecting line medial and lateral intercondylar tubercles [76]	Manual<br>• Midpoint most extreme points of medial and lateral malleoli [57]<br>• Midpoint cortical surface medial and lateral malleoli [22]<br>• Midpoint medial and lateral malleoli [3]<br>Automatic<br>• Midpoint line most prominent point TMM and distal tip FLM, with use of mean determinations of reference points [46, 47]<br>• Midpoint line most protruding point TMM and FLM [76]
		Intercondylar eminence center – AJC [4, 6, 18, 28, 39, 54, 72]	Manual<br>• Explicit method unknown [4, 6, 18, 28, 39, 54, 72]	Manual<br>• Center of all points on the tibial & fibular articular surface, defined by calculating the closest-point distance to the talus and subsequently only consider points below a user defined distance threshold [72]<br>• Center distal articular surfaces tibia and fibula [4, 28]<br>• Explicit method unknown [6, 18, 39, 54]
		Tibial center condyle – midpoint tibial plafond [42]	Manual<br>• Midpoint between the closest point on the tibial surface to the mean of four points on the medial condyle and the closest point on the tibial surface to the mean of four points on the lateral condyle [42]<br>• Four-fifth of the distance from the midpoint between the most posterior points of the medial and lateral tibial condyles to the	Manual<br>• Midpoint between midpoints of medial and lateral plafond of the distal tibial joint [42]<br>• Midpoint between the midpoint of the anterior ridge of the distal tibial joint and the midpoint of the posterior ridge of the distal tibial joint [42]

			midpoint between the most anterior points of the medial and lateral tibial condyles [42]	
		Midpoint Akagi's line – TIMA center [78, 87]	Manual<br>• Midpoint between medial third of tibial tubercle and posterior cruciate ligament insertion [78, 87]	Manual<br>• Midpoint medial and lateral malleoli [78, 87]
		Midpoint intercondylar ridge TP – midpoint ankle mortise [53, 56]	Manual<br>• Explicit method unknown [53, 56]	Manual<br>• Explicit method unknown [53, 56]
		Midpoint PTJ ML – midpoint tibia plafond [37]	Manual<br>• Midpoint of centers of best-fit circles to cortical edges of medial and lateral tibia plateaus [37]	Manual<br>• Geometric center of articular surface of tibia plafond [37]
		FAAD – AJC [88]	Automatic<br>• Intersection points of fitted line and bone model, with fitted line obtained by skeletonization algorithm [88]	Automatic<br>• Midpoint two largest curvature points proximal medial and lateral talus [88]
		Center x-axis – AJC [79]	Manual<br>• Midpoint centers best-fit circle cortical edge medial and lateral TP [79]	Manual<br>• Center articular surface talar trochlea [79]
		Proximal point proximal aTA – TIMA center [8]	Semi-automatic<br>• Proximal point of proximal aTA intersecting tibial surface. aTA obtained by center line cylinder fitting most distal cross-section proximal tibia and distal end of tibial tubercle [8]	Manual<br>• Point at 44% of the intermalleolar distance from the medial malleolus [8]
		ACL insertion – AJC [89]	Manual<br>• Explicit method unknown [89]	Manual<br>• Explicit method unknown [89]
PTJ ML	Landmark	Landmark pair	Medial	Lateral
		Tibial medial condyle center – tibial lateral condyle center [9, 16, 42, 46, 47, 78-80, 88]	Manual<br>• Center best-fit circle cortical edge medial TP [37, 79, 80]<br>• Deepest point medial tibia plateau [78]<br>• Explicit method unknown [9, 16]<br>• Closest point on the tibial surface to the mean of the midpoint of the medial tibial spine and the most medial, most anterior and most posterior points of the medial condyle [42]<br>Automatic<br>• Deepest point medial TP [46, 47, 88], with use of mean determinations of reference points [46, 47]	Manual<br>• Center best-fit circle cortical edge lateral TP [37, 79, 80]<br>• Deepest point lateral tibia plateau [78]<br>• Explicit method unknown [9, 16]<br>• Closest point on the tibial surface to the mean of the midpoint of the lateral tibial spine and the most lateral, most anterior and most posterior points of the lateral condyle [42]<br>Automatic<br>• Deepest point lateral TP [46, 47, 88], with use of mean determinations of reference points [46, 47]
		TMCM – TLCL [6, 19, 76, 80]	Manual<br>• Most cranial medial TP [6, 19]. Placed in cranial view, confirmed in medial view [19]<br>• Explicit method unknown [80]<br>Automatic<br>• Most medial prominence TP [76]	Manual<br>• Most cranial lateral TP [6, 19]. Placed in cranial view, confirmed in lateral view [19]<br>• Explicit method unknown [80]<br>Automatic<br>• Most lateral prominence TP [76]
	Calculated plane		Manual<br>• Best fit plane brush marked surface medial and lateral TP [24]<br>• Best fit plane 4 selected points as rhomboids on medial and lateral TP, using LSR [34, 71]<br>• Normal to the sagittal plane defined by (1) center of posterior intercondylar facet proximal to the enthesis of the posterior cruciate ligament, (2) proximal medial edge of the tibial tubercle and (3) midpoint of the medial and lateral talocrural facet margins [15]<br>• Coronal projection of best-fit plane best representing the AS of the tibial plateau, explicit method unknown [37]<br>• Frontal projection of least-squares plane fit through tibia plateau defined by 10 surface points [29]<br>Automatic<br>• Best fit plane 35 points selected on medial and lateral TP [32, 60, 67, 75], with three studies reporting algorithm-based measurements on corresponding bone, and mapped to chosen bone [32, 60, 67]	

PTJ AP	Landmark	Landmark pair<br>Tibial anterior condyle – tibial posterior condyle [42]	Anterior<br>Manual<br>• Midpoint between the most anterior points of the medial and lateral tibial condyles [42]	Posterior<br>Manual<br>• Midpoint between the most posterior points of the medial and lateral tibial condyles [42]
	Calculated plane		Manual<br>• Sagittal projection of best-fit plane best representing the AS of the tibial plateau, explicit method unknown [4, 37]<br>• Plane fit through vertices of most proximal 30 mm of the tibia bone model, projected to the sagittal plane. [72]<br>• Best fit plane marked surface medial and lateral TP, intersection with sagittal plane [25, 77]<br>Automatic<br>• Best fit plane 35 points selected on medial and lateral TP [32, 60, 67, 75] and intersection with sagittal plane, with three studies reporting algorithm-based measurements on corresponding bone, and mapped to chosen bone [32, 60, 67]	
PTJ APM	Landmark	Landmark pair<br>TMCA – TMCP [19, 33, 57, 58, 79, 92, 95]	Anterior<br>Manual<br>• Most cranial anterior [19, 33]. Placed in cranial view, confirmed in anterior view [19]<br>• Most prominent aspect of the anterior cortex of the medial compartment [58]<br>• Anterior point tibial outline sagittal plane intersection, by translation sagittal plane from mTA to center best fit circle medial tibial articular surface [79]<br>• Anterior lip of medial tibial plateau in sagittal plane through centroid of closed curve formed by tracing the edge of medial tibia plateau [57]<br>• Anterior edge TP, explicit method unknown [95]<br>• Anterior point of line tangent to articular surface medial TP, explicit method unknown [92]	Posterior<br>Manual<br>• Most cranial posterior [19, 33]. Placed in cranial view, confirmed in posterior view [19]<br>• Most prominent aspect of the posterior cortex of the medial compartment [58]<br>• Posterior point tibial outline sagittal plane intersection, by translation sagittal plane from mTA to center best fit circle medial tibial articular surface [79]<br>• Posterior lip of medial tibial plateau in sagittal plane through centroid of closed curve formed by tracing the edge of lateral tibia plateau [57]<br>• Posterior edge TP, explicit method unknown [95]<br>• Posterior point of line tangent to articular surface medial TP, explicit method unknown [92]
	Calculated plane		Manual<br>• Best fit plane brush marked surface medial TP [25, 77]<br>• Best fit plane brush marked elevated outermost rim or concavity medial TP, plane fitted using PCA with minimal squared distance [27]<br>Automatic<br>• Best fit plane 15 points medial articular surface TP, with measurements on first bone, and mapped to chosen bone [67]	
PTJ APL	Landmark	Landmark pair<br>TLCA – TLCP [19, 33, 57, 58, 79, 92, 95]	Anterior<br>Manual<br>• Most cranial anterior [19, 33]. Placed in cranial view, confirmed in anterior view [19]<br>• Most prominent aspect of the anterior cortex of the lateral compartment [58]<br>• Anterior point tibial outline sagittal plane intersection, by translation sagittal plane from mTA to center best fit circle lateral tibial articular surface [79]<br>• Anterior edge lateral TP, explicit method unknown [95]<br>• Anterior point of tangential line of tibial plateau in sagittal plane through centroid of closed curve formed by tracing the edge of the lateral tibia plateau [57]<br>• Anterior point of line tangent to articular surface lateral TP, explicit method unknown [92]<br>[57]	Posterior<br>Manual<br>• Most cranial posterior [19, 33]. Placed in cranial view, confirmed in posterior view [19]<br>• Most prominent aspect of the posterior cortex of the lateral compartment [58]<br>• Posterior point tibial outline sagittal plane intersection, by translation sagittal plane from mTA to center best fit circle lateral tibial articular surface [79]<br>• Posterior edge lateral TP, explicit method unknown [95]<br>• Posterior point of tangential line of tibial plateau in sagittal plane through centroid of closed curve formed by tracing the edge of the lateral tibia plateau [57]<br>• Posterior point of line tangent to articular surface lateral TP, explicit method unknown [92]
	Calculated plane		Manual<br>• Best fit plane brush marked surface lateral TP [25, 77]	

			• Best fit plane brush marked elevated outermost rim or concavity lateral TP, plane fitted using PCA with minimal squared distance [27]<br>Automatic<br>• Best fit plane maximum curvature points rim TP, by plane fitting algorithm relative to z-axis, starting at 25% of X-axis, at peak medial intercondylar tubercle [2]<br>• Best fit plane 15 points lateral articular surface TP, with measurements on first bone, and mapped to chosen bone [67]	
PTCA	Landmark	Landmark pair<br>TMCP – TLCP [9, 15, 19, 33, 35, 47, 80, 84, 93]	Medial<br>Manual<br>• Most dorsal cranial. Placed in cranial view, confirmed in dorsal view [19]<br>• Placed in cranial view, relative to mTA [84]<br>• Line tangent to posterior TP [9, 33, 35]<br>• Line tangent to posterior border tibial condyles [93]<br>• Line tangent to cross-sectional area at 10 mm below lateral tibial condylar joint surface [15]<br>• Explicit method unknown [80]<br>Automatic<br>• Explicit method unknown, with use of mean determinations of reference points [47]	Lateral<br>Manual<br>• Most dorsal cranial. Placed in cranial view, confirmed in dorsal view [19]<br>• Placed in cranial view, relative to mTA [84]<br>• Line tangent to posterior TP [9, 33, 35]<br>• Line tangent to posterior border tibial condyles [93]<br>• Line tangent to cross-sectional area at 10 mm below lateral tibial condylar joint surface [15]<br>• Explicit method unknown [80]<br>Automatic<br>• Explicit method unknown, with use of mean determinations of reference points [47]
DTJ ML	Landmark	Landmark pair<br>Tibial medial plafond – Tibial lateral plafond [42]	Medial<br>Manual<br>• Midpoint of the medial plafond of the distal tibial joint [42]	Lateral<br>Manual<br>• Midpoint of the lateral plafond of the distal tibial joint [42]
	Calculated line		Manual<br>Line bisecting the distal articular surface of tibial plafond [37]	
TIMA	Landmark	Landmark pair<br>TMM – FLM [15, 19, 33, 35, 57, 76]	Medial<br>Manual<br>• Most medial. Placed in caudal view, confirmed in medial view [19]<br>• Outermost point medial malleolus [33]<br>• Apex medial malleolus [15]<br>• Extreme point medial malleolus [57]<br>• Midpoint of articular surface medial malleolus [35]<br>Automatic<br>• Most protruding point TMM [76]	Lateral<br>Manual<br>• Most lateral. Placed in caudal view, confirmed in lateral view [19]<br>• Deepest point incisura fibularis tibiae [33]<br>• Apex lateral malleolus [15]<br>• Extreme point lateral malleolus [57]<br>• Midpoint of articular surface lateral malleolus [35]<br>Automatic<br>• Most protruding point FLM [76]
		TMM – distal FLM [47]	Automatic<br>• Most prominent point TMM, with use of mean determinations of reference points [47]	Automatic<br>• Most prominent (distal part) of the FLM, with use of mean determinations of reference points [47]
aTA	Landmark	Landmark pair<br>TAAP – TAAD [3, 19, 21, 33, 42, 45, 77]	Proximal<br>Manual<br>• Midpoint at 1/3 shaft length [19, 21, 33]. Points placed in dorsal and ventral view, medial view for midpoint of the connecting line [19]<br>• Center point medullary cavity 5 cm below tibial tubercle [45]<br>• Tibial medullary cavity midpoint at distal end of tibial tuberosity [3]<br>Semi-automatic<br>• Centre of sphere fitting marked cortex at 1/3 shaft length [77]<br>Automatic<br>• Midpoint of the tibial transverse cross-section at 75% of distance along the tibial axis [42]	Distal<br>Manual<br>• Midpoint at 2/3 shaft length [19, 21, 33]. Points placed in dorsal and ventral view, medial view for midpoint of the connecting line [19]<br>• Center point medullary cavity 5 cm above ankle mortise [45]<br>• 10cm below defined proximal landmark [3]<br>Semi-automatic<br>• Centre of sphere fitting marked cortex at 2/3 shaft length [77]<br>Automatic<br>• Midpoint of the tibial transverse cross-section at 15% of distance along the tibial axis [42]
		TAAP – Midpoint tibial plafond area [24, 25]	Manual	Manual

			• Projected point on TP, based on center points proximal tibia [24, 25]	• Spherical center fitted to tibial plafond and medial and lateral malleolar articular surfaces [24, 25]
		Intercondylar eminence center – parallel to posterior shaft [92]	Manual<br>• Explicit method unknown [92]	Manual<br>• Parallel to posterior tibial shaft in sagittal plane, and medial and lateral tibial shaft in coronal plane [92]
		Intercondylar eminence center – midpoint tibial plafond area [9]	Manual<br>• Explicit method unknown [9]	Manual<br>• Explicit method unknown [9]
	Calculated line		Automatic<br>• Best fit curve, spherical center at 2/8 to 7/8 tibia length [76]<br>• Skeletonization algorithm, resulting in curve, with fitted line resulting in TAAP and TAAD intersection points with bone model [88]	
paTA	Landmark	Landmark pair<br>TAAP1 – TAAP2 [42, 77, 79, 95]	Proximal<br>Semi-automatic<br>• Midpoint tibial cross-section at 15% distance along tibial axis [42]<br>• Center of sphere fit to marked cortex at 5 cm from proximal tibia [77]<br>Manual<br>• Midpoint, anterior cortex or posterior cortex 5 cm distal to knee joint [95]<br>• Most anterior point tibial cortex 5 cm distal to knee joint line on sagittal plane [79]	Distal<br>Semi-automatic<br>• Midpoint tibial cross-section at 30% distance along tibial axis [42]<br>• Center of sphere fit to marked cortex at 10 cm from proximal tibia [77]<br>Manual<br>• Midpoint, anterior cortex or posterior cortex 15 cm distal to knee joint [95]<br>• Most anterior point tibial cortex 15 cm distal to knee joint line on sagittal plane [79]
	Calculated line		Automatic<br>• LSR through center points of axial cross sections of the tibial shaft from 15 cm distal from articular surface with 5 mm steps proximal until cross-section area exceeds 1.6-fold the starting cross-section area [27]<br>• Bisecting line of the proximal tibia shaft on true lateral view, explicit method unknown [63]	
	Best fit geometrical shape		Semi-automatic<br>• Axis of best-fit cylinder between most distal cross-section proximal tibia and distal end of tibial tubercle [8]	

Abbreviations: AJC, ankle joint center; aTA, anatomical tibial axis; CPD, closest point distance; DTJ ML, distal medial-lateral tibial joint orientation; FAAD, femoral anatomical axis distal; FLM, fibular lateral malleolus; KJC, knee joint center; LSR, least square regression; mTA, mechanical tibial axis; paTA, partial anatomical tibial axis; PCA, principal component analysis; PTCA, posterior tibial condylar axis; PTJ AP, proximal anterior-posterior tibial joint orientation; PTJ APL, proximal anterior-posterior lateral tibial joint orientation; PTJ APM, proximal anterior-posterior medial tibial joint orientation; PTJ ML, proximal medial-lateral tibial joint orientation; TAAD, tibial anatomical axis distal; TAAP, tibial anatomical axis proximal; TIMA, tibial intermalleolar axis; TP, tibial plateau; TLCA, anterior lateral tibial condyle; TLCL, most lateral lateral tibial condyle; TLCP, posterior lateral tibial condyle; TMCA, anterior medial tibial condyle; TMCM, medial medial tibial condyle; TMCP, posterior medial tibial condyle.

Table 6. Overview of methods to derive leg axes from a 3D bone model. For the leg, these methods were grouped into one category: landmark method (a line between two landmark points). The explicit method per study is described by a landmark pair followed by reported methods for each of the landmarks.

Axis	Category	Method		
mLA	Landmark	Landmark pair	Proximal	Distal
		FHC – midpoint tibial plafond area [19, 42]	Manual<br>• Midpoint of line in medial view, by connecting midpoint femoral head posterior and anterior view [19]<br>Semi-automatic: spherical center fitted to FH<br>• Explicit method unknown [42]	Manual<br>• Placed and confirmed in caudal view [19]<br>• Midpoint between midpoints of medial and lateral plafond of the distal tibial joint [42]
		FHC – AJC [6, 54, 88]	Manual<br>• Explicit method unknown [6, 54]<br>Semi-automatic: spherical center fitted to FH<br>• Selected points on femoral head, followed by an unknown method [88]	Manual<br>• Explicit method unknown [6, 54]<br>Automatic<br>• Midpoint two largest curvature points proximal medial and lateral talus [88]

Abbreviations: AJC, ankle joint center; FHC, femoral head center; mLA, mechanical leg axis.

Table 7. Overview of used definitions for a complete femoral coordinate system (CS). A coordinate system was defined to be complete if implicitly or explicitly a coordinate system with 3 orthogonal axes could be derived. Femoral CSs were defined using all three possible combinations of two axes.[a] The third axis follows from these definitions and is therefore not included. Medial-lateral axes were categorized as the x-axis, anterior-posterior axes were categorized as the y-axis, and cranial-caudal axes were categorized as the z-axis [20].

Axis pair	Axis	Category	Method	Axis	Category	Method
z-x [1, 6, 31, 38, 45, 49, 52, 55, 56, 61, 62, 66, 70, 74, 84]	z	Landmark	mFA [1, 6, 38, 45, 49, 52, 55, 56, 62, 66, 70, 74, 84]	x	Landmark	FCA [38, 62, 70, 84]<br>FEA [1, 45, 49, 52, 55, 56, 66]<br>PFCA [74]
					Best fit geometrical shape	FCA (best-fit cylinder both femoral condyles) [6, 62, 70]
	z	Calculated line	The inertial axis defined along the femoral diaphyseal shaft [61]	x	Best fit geometrical shape	FCA (best-fit cylinder both femoral condyles) [61]
	z	Calculated line	Projection line of femoral shaft on sagittal plane [31]	x	Best fit geometrical shape	FCA (co-axis of best-fit cylinders medial and lateral femoral condyles) [31]
x-z [17]	x	Landmark	FEA [17]<br>PFCA [17]	z	Landmark	mFA [17]<br>aFA [17]
x-y [17, 30]	x	Landmark	PFCA [17]	y	Calculated plane	Normal to femoral tabletop plane [17]
			FCA [30]		Calculated line	Normal to plane defined by x-axis and FH center [30]
y-x [64]	y	Calculated plane	Normal to tabletop plane defined by three posterior points femur [64]	x	Landmark	DFJ [64]

Abbreviations: DFJ, distal femoral joint orientation; FCA, femoral condylar axis; FEA, femoral epicondylar axis; mFA, mechanical femoral axis; PFCA, posterior femoral condylar axis. [a] One study [35] fully relied on principal component analysis of the distal femur to establish complete coordinate systems, and could therefore not be included in this table.

Table 8. Overview of used definitions for a complete tibial coordinate system (CS). A coordinate system was defined to be complete if implicitly or explicitly a coordinate system with 3 orthogonal axes could be derived. Tibial CSs were defined using all three possible combinations of two axes.[a] The third axis follows from these definitions and is therefore not included. Medial-lateral axes were categorized as the x-axis, anterior-posterior axes were categorized as the y-axis, and cranial-caudal axes were categorized as the z-axis [20].

Axis pair	Axis	Category	Method	Axis	Category	Method
z-x [2, 3, 8, 11, 37, 38, 57, 62, 70, 84, 85, 90, 92]	z	Landmark	mTA [3, 8, 37, 57, 62, 70, 84, 90]	x	Landmark	PTJ ML [3, 8, 37, 70, 84, 90]
						FCA [62]
						TIMA [57]
					Calculated line	Principal inertial axis of a bone layer containing the tibial plateau [70]
					Best fit geometrical shape	Major axis contoured ellipse fitting tibial plateau [70]
			aTA [92]		Landmark	PTJ ML [92]
	z	Calculated line	Smallest eigenvalue of principal component analysis tibia [2, 85] or tibia and fibula combined [38]	x	Best fit geometrical shape	Major axis of contoured ellipse fitting tibial plateau perpendicular to y-axis [2, 38]
					Landmark	Axis perpendicular to y-axis pointing medially to most distal point of polygon mesh (medial malleolus) [85]
	z	Calculated plane	Normal to plane fit through three points on tibial plateau [11]	x	Landmark	PTJ ML [11]
z-y [15, 40, 43, 54, 73, 79, 89]	z	Landmark	mTA [15, 54, 73, 79, 89]	y	Landmark	Akagi's line[b] [15, 40, 43, 73, 79, 89]
		Calculated plane	Normal to best fit plane articular surface medial tibial plateau [40, 63]			Midpoint intercondylar spine – Medial midpoint tibial insertion of PCL [54]
			Normal to best fit plane articular surface medial and lateral tibial plateaus [43]			
y-x [31, 61]	y	Calculated line	AP center line of rectangle fitted the tibial plateau at the level of the fibular apex [31]	x	Calculated line	ML center line of rectangle fitted around the tibial plateau at the level of the fibular apex [31]
	y		Short inertial axis of the tibial plateau defined as the largest cross-sectional area of the proximal tibia [61]	x		Long inertial axis of the tibial plateau defined as the largest cross-sectional area of the proximal tibia [61]
x-z [11]	x	Landmark	PFCA [11]	z	Calculated plane	Normal to plane through three points on tibial plateau [11]

Abbreviations: aTA, anatomical tibial axis; AP, anterior-posterior; ML, medial-lateral; mTA, mechanical tibial axis; PFCA, posterior femoral condylar axis; PTJ, proximal tibial joint orientation.

[a] One study [35] fully relied on principal component analysis of proximal tibia to establish complete coordinate systems, and could therefore not be included in this table.

[b] Akagi's line was always defined as a line connecting (1) the insertion of the posterior cruciate ligament and (2) the medial border of the tibial tuberosity.

Table 9. Overview of the inter- and intra-observer reliability of leg alignment parameters per study. The number of observers is given, and the leg alignment parameters were defined by two lines in the coronal, sagittal, or axial planes. ICC values between 0-0.50, 0.50-0.75, 0.75-0.90, and 0.90-1.00 indicate poor, moderate, good, and excellent reliability, respectively [41].

Study	Observers	Axes or joint orientations		Plane	Inter (ICC)	Intra (ICC)
Amirtharaj [2]	3	aTA	PTJ APL	Sagittal	0.998	0.999
Chalmers [8]	2	mTA	aTA	Sagittal	0.87	NA
Fürmetz [19]	3	mFA	mTA	Coronal	NA	0.96, 95% CI [0.942, 0.998]
				Sagittal	NA	0.99, 95% CI [0.942, 0.998]
		aFA	NFA	Coronal	NA	0.94, 95% CI [0.448, 0.992]
		aFA	HJ	Coronal (medial)	NA	0.96, 95% CI [0.782, 0.995]
		aFA	HJ	Coronal (lateral)	NA	0.96, 95% CI [0.799, 0.994]
		mFA	DFJ	Coronal	NA	0.99, 95% CI [0.968, 0.999]
		mTA	PTJ ML	Coronal	NA	0.98, 95% CI [0.869, 0.997]
		mTA	PTJ APM	Sagittal	NA	0.80, 95% CI [0.256, 0.969]
		mTA	PTJ APL	Sagittal	NA	0.90, 95% CI [0.583, 0.985]
		NFA	PFCL	Axial	NA	0.92, 95% CI [0.410, 0.990]
		PTCA	TIMA	Axial	NA	0.69, 95% CI [0.040, 0.950]
Hancock [22]	3	mFA	FEA	NR	0.83	NA
		mFA	FCA	NR	0.56	NA
		mTA	FEA	NR	0.86	NA
		mTA	FCA	NR	0.70	NA
Ho [25]	2	aTA	PTJ AP	Sagittal (for 2 CS)	0.93	NA
			PTJ APM	Sagittal (for 2 CS)	0.93	NA
			PTJ APL	Sagittal (for 2 CS)	0.93	NA
Ho [24]	2	Proximal aTA	PTJ ML	Coronal (for 2 CS)	NA	0.96
Hoch [27]	2	Proximal aTA	PTJ APM (plateau)	Sagittal	0.909	NA
		Proximal aTA	PTJ APM (rim)	Sagittal	0.987	NA
		Proximal aTA	PTJ APL (plateau)	Sagittal	0.918	NA
		Proximal aTA	PTJ APL (rim)	Sagittal	0.893	NA
Jud [36]	2	mFA	mTA	Coronal	0.997, 95% CI [0.957, 0.999]	0.995, 95% CI [0.960, 0.998]
Jud [34]	2	mFA	mTA	Coronal	0.988, 95% CI [0.981,0.992]	NA
		DFJ	PTJ ML	Coronal	0.844, 95% CI [0.743,0.903]	NA
Jud [35]	2	PTCA	TIMA	Axial	0.980, 95% CI [0.784, 0.997]<br>0.988, 95% CI [0.940, 0.998]<br>0.883, 95% CI [0.418, 0.979]	NA
Kuiper [42]	1	Proximal aTA	PTJ AP	Sagittal	NA	0.93, 95% CI [0.87, 0.96]
		aTA	PTJ AP	Sagittal	NA	0.95, 95% CI [0.91, 0.98]
		mTA	PTJ AP	Sagittal	NA	0.93, 95% CI [0.87, 0.96]
		mFA	mTA	Coronal	NA	0.99, 95% CI [0.99, 1.00]
		DFJ	PTJ ML	Coronal	NA	0.97, 95% CI [0.94,0.98]
		mFA	DFJ	Coronal	NA	0.96, 95% CI [0.92, 0.98]
		mTA	DTJ ML	Coronal	NA	0.76, 95% CI [0.58, 0.86]
		mFA	HJ	Coronal	NA	0.99, 95% CI [0.98, 0.99]
		mTA	PTJ ML	Coronal	NA	0.99, 95% CI [0.98, 0.99]
León-Muñoz [46]	1	mFA	mTA	Coronal	NA	0.987, 95% CI [0.984, 0.99]
		mFA	DFJ	Coronal	NA	0.972, 95% CI [0.964, 0.978]
		mTA	PTJ ML	Coronal	NA	0.975, 95% CI [0.967,0.98]
Liu [55]	3	mFA	DFJ	Coronal	NA	> 0.8
Liu [51]	2	FEA (anatomical)	FEA (surgical)	Axial	0.906, 95% CI [0.868-0.934]	0.926, 95% CI [0.896-0.948]
		PFCA	FEA (surgical)	Axial	0.970, 95% CI [0.957-0.979]	0.927, 95% CI [0.892-0.946]
Liu [52]	2	PFCA	FEA (surgical)	Axial	0.964, 95% CI [0.950-0.974]	0.965, 95% CI [0.952-0.974]

Moon [63]	2	Proximal aTA	PTJ ML	Coronal	NA	0.972 – 0.995
		Proximal aTA	PTJ APM	Sagittal	NA	0.972 – 0.995
Roth [71]	2	DFJ	PTJ ML	Coronal	0.961, 95% CI [0.902,0.984], p<0.001	NA
		mFA	mTA	Coronal	0.961, 95% CI [0.902,0.984], p<0.001	NA
Sasaki [73]	2	mFA	mTA	Coronal	0.97	0.98 & 0.99
		mFA	DFJ	Coronal	0.97	0.98 & 0.99
		mTA	PTJ ML	Coronal	0.97	0.98 & 0.99
		DFJ	PTJ ML	Coronal	0.97	0.98 & 0.99
Teng [79]	2	Proximal aTA	PTJ ML	Coronal	0.91 – 0.99	0.91 – 0.99
		mTA	PTJ APM	Sagittal	0.91 – 0.99	0.91 – 0.99
		mTA	PTJ APL	Sagittal	0.91 – 0.99	0.91 – 0.99
Yamagami [89]	2	mTA	PTJ APM	Sagittal	0.718	0.838
Zhang [93]	2	mFA	mTA	Coronal	>0.8	>0.8
			PTJ ML	Coronal	0.727	0.813
Zhang [95]	2	Proximal aTA/(anterior/posterior) tibial cortex line	PTJ APM/PTJ APL	Sagittal	0.77 – 0.91	0.77 – 0.91

Abbreviations: aFA, anatomical femoral axis; AP(M/L), anterior-posterior (medial/lateral); aTA, anatomical tibial axis; DFJ, distal femoral joint orientation; DTJ; distal tibial joint orientation; HJ, hip joint orientation; mFA, mechanical femoral axis; ML, medial-lateral; mTA, mechanical tibial axis; NA, not applicable; NFA, neck femur axis; NR, not reported; PFCA, posterior femoral condylar axis; PTCA, posterior tibial condylar axis; PTJ, proximal tibial joint orientation; TIMA, tibial intermalleolar axis.

REFERENCES

1. Adachi T, Kato Y, Kiyotomo D, Kawamukai K, Takazawa S, Suzuki T, et al. (2023) Accuracy Verification of Four-Dimensional CT Analysis of Knee Joint Movements: A Pilot Study Using a Knee Joint Model and Motion-Capture System. Cureus 15:e35616; https://doi.org/10.7759/cureus.35616
2. Amirtharaj MJ, Hardy BM, Kent RN, Nawabi DH, Wickiewicz TL, Pearle AD, et al. (2018) Automated, accurate, and three-dimensional method for calculating sagittal slope of the tibial plateau. Journal of biomechanics 79:212-217; https://doi.org/10.1016/J.JBIOMECH.2018.07.047
3. An HM, Wen JX, Gu W, Chen JY, Chai W, Li R (2024) Discrepancies in Sagittal Alignment of the Lower Extremity Among Different Brands of Robotic Total Knee Arthroplasty Systems. The Journal of Arthroplasty; https://doi.org/10.1016/j.arth.2024.03.029
4. Arn Roth T, Jokeit M, Sutter R, Vlachopoulos L, Fucentese SF, Carrillo F, et al. (2024) Deep-learning based 3D reconstruction of lower limb bones from biplanar radiographs for preoperative osteotomy planning. International Journal of Computer Assisted Radiology and Surgery; https://doi.org/10.1007/s11548-024-03110-5
5. Berryman F, Pynsent P, McBryde C (2014) A semi-automated method for measuring femoral shape to derive version and its comparison with existing methods. International Journal for Numerical Methods in Biomedical Engineering 30:1314-1325; https://doi.org/10.1002/CNM.2659
6. Brunner J, Jörgens M, Weigert M, Kümpel H, Degen N, Fuermetz J (2023) Significant changes in lower limb alignment due to flexion and rotation-a systematic 3D simulation of radiographic measurements. Knee Surgery, Sports Traumatology, Arthroscopy 31:1483-1490; https://doi.org/10.1007/s00167-022-07302-x
7. Chalmers BP, Borsinger TM, Quevedo Gonzalez FJ, Vigdorchik JM, Haas SB, Ast MP (2023) Referencing the center of the femoral head during robotic or computer-navigated primary total knee arthroplasty results in less femoral component flexion than the traditional intramedullary axis. The Knee 44:172-179; https://doi.org/10.1016/j.knee.2023.08.006
8. Chalmers BP, Quevedo-Gonzalez F, Gausden EB, Jerabek SA, Haas SB, Ast MP (2022) Posterior Tibial Slope in Computer-Navigated Total Knee Arthroplasty: The Transmalleolar Sagittal Axis Underestimates Slope Compared to Traditional Intramedullary Axis. The Journal of Arthroplasty 37:S207-S210; https://doi.org/10.1016/j.arth.2022.02.085
9. Cho BW, Lee T-H, Kim S, Choi C-H, Jung M, Lee KY, et al. (2021) Evaluation of the reliability of lower extremity alignment measurements using EOS imaging system while standing in an even weight-bearing posture. Scientific Reports 11:22039-22039; https://doi.org/10.1038/s41598-021-01646-z
10. Cho HJ, Kwak DS, Kim IB (2015) Morphometric evaluation of Korean femurs by geometric computation: Comparisons of the sex and the population. BioMed Research International 2015; https://doi.org/10.1155/2015/730538
11. Chung JH, Choi CH, Kim SH, Kim SJ, Suk YJ, Jung M (2022) Effect of the sagittal osteotomy inclination angle on the posterior tibial slope change in high tibial osteotomy: three-dimensional simulation study. Scientific Reports 12:19254; https://doi.org/10.1038/s41598-022-23412-5
12. Citak M, Oszwald M, O'Loughlin PF, Citak M, Kendoff D, Hüfner T, et al. (2010) Three-dimensional measurement of femoral antetorsion: comparison to a conventional radiological method. Archives of orthopaedic and trauma surgery 130:513-518; https://doi.org/10.1007/S00402-009-0923-8
13. Degen N, Sass J, Jalali J, Kovacs L, Euler E, Prall WC, et al. (2020) Three-dimensional assessment of lower limb alignment: Reference values and sex-related differences. The Knee 27:428-435; https://doi.org/10.1016/J.KNEE.2019.11.009
14. Eckhoff D, Hogan C, DiMatteo L, Robinson M, Bach J (2007) Difference between the epicondylar and cylindrical axis of the knee. Clinical orthopaedics and related research 461:238-244; https://doi.org/10.1097/BLO.0B013E318112416B
15. Enomoto H, Nakamura T, Waseda A, Niki Y, Toyama Y, Suda Y (2013) A novel and reproducible reference axis for distal tibial axial rotation. The Journal of arthroplasty 28:788-791; https://doi.org/10.1016/j.arth.2012.11.005
16. Factor S, Gurel R, Dan D, Benkovich G, Sagi A, Abialevich A, et al. (2024) Validating a Novel 2D to 3D Knee Reconstruction Method on Preoperative Total Knee Arthroplasty Patient Anatomies. Journal of Clinical Medicine 13; https://doi.org/10.3390/jcm13051255
17. Fischer MCM, Grothues S, Habor J, de la Fuente M, Radermacher K (2020) A robust method for automatic identification of femoral landmarks, axes, planes and bone coordinate systems using surface models. Scientific Reports 10:20859; https://doi.org/10.1038/s41598-020-77479-z
18. Flury A, Hodel S, Hasler J, Hooman E, Fucentese SF, Vlachopoulos L (2022) The winking sign is an indicator for increased femorotibial rotation in patients with recurrent patellar instability. Knee Surgery, Sports Traumatology, Arthroscopy 30:3651-3658; https://doi.org/10.1007/s00167-022-06971-y

19. Fürmetz J, Sass J, Ferreira T, Jalali J, Kovacs L, Mück F, et al. (2019) Three-dimensional assessment of lower limb alignment: Accuracy and reliability. The Knee 26:185-193; https://doi.org/10.1016/J.KNEE.2018.10.011
20. Grood ES, Suntay WJ (1983) A joint coordinate system for the clinical description of three-dimensional motions: applications to the knee. Journal of biomechanical engineering; https://doi.org/10.1115/1.3138397
21. Hanada M, Hotta K, Matsuyama Y (2020) A computer simulation study for preserving the tibial posterior slope in open-wedge high tibial osteotomy. European Journal of Orthopaedic Surgery & Traumatology 30:1285-1291; https://doi.org/10.1007/S00590-020-02703-5
22. Hancock CW, Winston MJ, Bach JM, Davidson BS, Eckhoff DG (2013) Cylindrical axis, not epicondyles, approximates perpendicular to knee axes. Clinical orthopaedics and related research 471:2278-2283; https://doi.org/10.1007/S11999-013-2864-3
23. Hartel MJ, Petersik A, Schmidt A, Kendoff D, Nüchtern J, Rueger JM, et al. (2016) Determination of femoral neck angle and torsion angle utilizing a novel three-dimensional modeling and analytical technology based on CT datasets. PLoS ONE 11; https://doi.org/10.1371/JOURNAL.PONE.0149480
24. Ho JPY, Merican AM, Ayob KA, Sulaiman SH, Hashim MS (2021) Tibia vara in Asians: Myth or fact? Verification with three-dimensional computed tomography. Journal of orthopaedic surgery (Hong Kong) 29; https://doi.org/10.1177/2309499021992618
25. Ho JPY, Merican AM, Hashim MS, Abbas AA, Chan CK, Mohamad JA (2017) Three-Dimensional Computed Tomography Analysis of the Posterior Tibial Slope in 100 Knees. The Journal of arthroplasty 32:3176-3183; https://doi.org/10.1016/J.ARTH.2017.04.060
26. Hoch A, Hasler J, Schenk P, Ackermann J, Ebert L, Fürnstahl P, et al. (2022) Registration based assessment of femoral torsion for rotational osteotomies based on the contralateral anatomy. BMC Musculoskeletal Disorders 23:962; https://doi.org/10.1186/s12891-022-05941-2
27. Hoch A, Jud L, Roth T, Vlachopoulos L, Fürnstahl P, Fucentese SF (2020) A real 3D measurement technique for the tibial slope: differentiation between different articular surfaces and comparison to radiographic slope measurement. BMC Musculoskeletal Disorders 21; https://doi.org/10.1186/S12891-020-03657-9
28. Hodel S, Arn-Roth T, Haug F, Carillo F, Vlachopoulos L, Fucentese SF, et al. (2024) The influence of the weight-bearing state on three-dimensional (3D) planning in lower extremity realignment – analysis of novel vs. state-of-the-art planning approaches. Archives of Orthopaedic and Trauma Surgery 144:1989-1996; https://doi.org/10.1007/s00402-024-05289-3
29. Hodel S, Hasler J, Roth TA, Flury A, Sutter C, Fucentese SF, et al. (2024) Validation of a Three-Dimensional Weight-Bearing Measurement Protocol for Medial Open-Wedge High Tibial Osteotomy. Journal of Clinical Medicine 13; https://doi.org/10.3390/jcm13051280
30. Huan W, Mochizuki T, Tanifuji O, Kawashima H (2023) Variability of functional knee phenotype for coronal alignment in advanced varus knee osteoarthritis in the Japanese population. Knee Surgery, Sports Traumatology, Arthroscopy 31:1451-1461; https://doi.org/10.1007/s00167-022-07248-0
31. Ikuta F, Yoneta K, Miyaji T, Kidera K, Yonekura A, Osaki M, et al. (2020) Association between stages of medial compartment osteoarthritis and three-dimensional knee alignment in the supine position: A cross-sectional study. Journal of clinical orthopaedics and trauma 11:S130-S136; https://doi.org/10.1016/J.JCOT.2019.10.011
32. Jacquet C, Laumonerie P, LiArno S, Faizan A, Sharma A, Dagneaux L, et al. (2019) Contralateral preoperative templating of lower limbs' mechanical angles is a reasonable option. Knee Surgery, Sports Traumatology, Arthroscopy 28:1445-1451; https://doi.org/10.1007/S00167-019-05524-0
33. Jörgens M, Keppler AM, Ahrens P, Prall WC, Bergstraesser M, Bachmeier AT, et al. (2022) 3D osteotomies—improved accuracy with patient-specific instruments (PSI). European Journal of Trauma and Emergency Surgery; https://doi.org/10.1007/s00068-022-02060-4
34. Jud L, Roth T, Fürnstahl P, Vlachopoulos L, Sutter R, Fucentese SF (2020) The impact of limb loading and the measurement modality (2D versus 3D) on the measurement of the limb loading dependent lower extremity parameters. BMC Musculoskeletal Disorders 21:1-9; https://doi.org/10.1186/S12891-020-03449-1
35. Jud L, Singh S, Tondelli T, Furnstahl P, Fucentese SF, Vlachopoulos L (2020) Combined Correction of Tibial Torsion and Tibial Tuberosity-Trochlear Groove Distance by Supratuberositary Torsional Osteotomy of the Tibia. American Journal of Sports Medicine 48:2260-2267; https://doi.org/10.1177/0363546520929687
36. Jud L, Trache T, Tondelli T, Fürnstahl P, Fucentese SF, Vlachopoulos L (2019) Rotation or flexion alters mechanical leg axis measurements comparably in patients with different coronal alignment. Knee Surgery, Sports Traumatology, Arthroscopy 28:3128-3134; https://doi.org/10.1007/S00167-019-05779-7
37. Jung SH, Jung M, Chung K, Kim S, Park J, Lee JH, et al. (2024) Factors Causing Unintended Sagittal and Axial Alignment Changes in High Tibial Osteotomy: Comparative 3-Dimensional Analysis of Simulation and Actual Surgery. American Journal of Sports Medicine 52:1543-1553; https://doi.org/10.1177/03635465241241539

38. Kai S, Sato T, Koga Y, Omori G, Kobayashi K, Sakamoto M, et al. (2014) Automatic construction of an anatomical coordinate system for three-dimensional bone models of the lower extremities--pelvis, femur, and tibia. Journal of biomechanics 47:1229-1233; https://doi.org/10.1016/J.JBIOMECH.2013.12.013
39. Kawahara S, Hara D, Murakami K, Hamai S, Akasaki Y, Tsushima H, et al. (2022) Smaller femoral neck anteversion in varus knees than in healthy and valgus knees. Clinical Anatomy 35:1044-1050; https://doi.org/10.1002/ca.23862
40. Kim JH, Kim HY, Lee DH (2020) Opening gap width influences distal tibial rotation below the osteotomy site following open wedge high tibial osteotomy. PLOS ONE 15:e0227969-e0227969; https://doi.org/10.1371/JOURNAL.PONE.0227969
41. Koo TK, Li MY (2016) A Guideline of Selecting and Reporting Intraclass Correlation Coefficients for Reliability Research. Journal of Chiropractic Medicine 15:155-163; https://doi.org/10.1016/J.JCM.2016.02.012
42. Kuiper RJA, Seevinck PR, Viergever MA, Weinans H, Sakkers RJB (2023) Automatic Assessment of Lower-Limb Alignment from Computed Tomography. The Journal of Bone and Joint Surgery 105:700-712; https://doi.org/10.2106/jbjs.22.00890
43. Lee BH, Ha CW, Moon SW, Chang M, Kim HY, Park SH, et al. (2017) Three-dimensional relationships between secondary changes and selective osteotomy parameters for biplane medial open-wedge high tibial osteotomy. The Knee 24:362-371; https://doi.org/10.1016/J.KNEE.2016.11.010
44. Lei K, Liu LM, Luo JM, Ma C, Feng Q, Yang L, et al. (2022) Could surgical transepicondylar axis be identified accurately in preoperative 3D planning for total knee arthroplasty? A reproducibility study based on 3D-CT. Arthroplasty 4:46; https://doi.org/10.1186/s42836-022-00147-2
45. Lei K, Liu LM, Xiang Y, Chen X, Fan HQ, Peng Y, et al. (2020) Clinical value of CT-based patient-specific 3D preoperative design combined with conventional instruments in primary total knee arthroplasty: a propensity score-matched analysis. Journal of Orthopaedic Surgery and Research 15:591; https://doi.org/10.1186/s13018-020-02123-5
46. León-Muñoz VJ, López-López M, Martínez-Martínez F, Santonja-Medina F (2020) Comparison of weight-bearing full-length radiographs and computed-tomography-scan-based three-dimensional models in the assessment of knee joint coronal alignment. The Knee 27:543-551; https://doi.org/10.1016/J.KNEE.2019.11.017
47. León-Muñoz VJ, Manca S, López-López M, Martínez-Martínez F, Santonja-Medina F (2021) Coronal and axial alignment relationship in Caucasian patients with osteoarthritis of the knee. Scientific Reports 11:1-8; https://doi.org/10.1038/s41598-021-87483-6
48. León-Muñoz VJ, Parrinello A, Galloni G, Lisón-Almagro AJ, López-López M, Martínez-Martínez F, et al. (2022) Reliability of the posterior condylar offset. Journal of Orthopaedic Research 40:1794-1800; https://doi.org/10.1002/jor.25205
49. Li C, Chen J, Yang Y, Jin Y, Wang C, Tsai TY, et al. (2022) Asymmetry of Posterior Condyles in Resection Plane and Axial Curvature for Total Knee Arthroplasty. Orthopaedic Surgery 14:3340-3348; https://doi.org/10.1111/os.13529
50. Li Z, Liu G, Tian R, Kong N, Li Y, Li Y, et al. (2021) The patellofemoral morphology and the normal predicted value of tibial tuberosity-trochlear groove distance in the Chinese population. BMC Musculoskeletal Disorders 22:1-13; https://doi.org/10.1186/S12891-021-04454-8/TABLES/3
51. Liu K, Liu X, Guan Y, Ma H, Fu D, Fan Z (2023) Accuracy and reproducibility analysis of different reference axes for femoral prosthesis rotation alignment in TKA based on 3D CT femoral model. BMC Musculoskeletal Disorders 24; https://doi.org/10.1186/s12891-023-06781-4
52. Liu K, Liu Y, Fan Z, Fu D (2023) Accuracy and reproducibility of two-dimensional computed tomography-based positioning of femoral component rotational alignment in preoperative planning for total knee arthroplasty. Journal of Orthopaedic Surgery and Research 18; https://doi.org/10.1186/s13018-023-04466-1
53. Liu L, Lei K, Chen X, Fan H, Yang L, Guo L (2022) Is Valgus Cut Angle Based on Radiographic Measurements in Total Knee Arthroplasty Really Inaccurate? A Comparison of Two- and Three-Dimensional Measurements. Journal of Knee Surgery 35:1563-1570; https://doi.org/10.1055/s-0041-1728785
54. Liu L, Lei K, Du D, Lin Y, Pan Z, Guo L (2024) Functional knee phenotypes appear to be more suitable for the Chinese OA population compared with CPAK classification: A study based on 3D CT reconstruction models. Knee Surgery, Sports Traumatology, Arthroscopy 32:1264-1274; https://doi.org/10.1002/ksa.12130
55. Liu L, Lei K, Guo L, Chen X, Yang P, Fu D, et al. (2022) Surgical Transepicondylar Axis Is Not a Reliable Reference when there Was Lateral Femoral Bowing. Orthopaedic Surgery 14:3209-3215; https://doi.org/10.1111/os.13545
56. Liu LM, Lei K, Chen X, Fu DJ, Yang P, Yang L, et al. (2021) Proximal external femoral torsion increases lateral femoral shaft bowing: a study based on 3D CT reconstruction models. Knee Surgery, Sports Traumatology, Arthroscopy 1-9; https://doi.org/10.1007/S00167-021-06753-Y

57. Ma QL, Lipman JD, Cheng CK, Wang XN, Zhang YY, You B (2017) A Comparison Between Chinese and Caucasian 3-Dimensional Bony Morphometry in Presimulated and Postsimulated Osteotomy for Total Knee Arthroplasty. The Journal of arthroplasty 32:2878-2886; https://doi.org/10.1016/j.arth.2017.03.069
58. Meier M, Janssen D, Koeck FX, Thienpont E, Beckmann J, Best R (2020) Variations in medial and lateral slope and medial proximal tibial angle. Knee Surgery, Sports Traumatology, Arthroscopy 2020 29:3 29:939-946; https://doi.org/10.1007/S00167-020-06052-Y
59. Meric G, Gracitelli GC, Aram LJ, Swank ML, Bugbee WD (2015) Variability in Distal Femoral Anatomy in Patients Undergoing Total Knee Arthroplasty: Measurements on 13,546 Computed Tomography Scans. The Journal of Arthroplasty 30:1835-1838; https://doi.org/10.1016/j.arth.2015.04.024
60. Micicoi G, Jacquet C, Sharma A, LiArno S, Faizan A, Kley K, et al. (2020) Neutral alignment resulting from tibial vara and opposite femoral valgus is the main morphologic pattern in healthy middle-aged patients: an exploration of a 3D-CT database. Knee Surgery, Sports Traumatology, Arthroscopy 2020 29:3 29:849-858; https://doi.org/10.1007/S00167-020-06030-4
61. Miranda DL, Rainbow MJ, Leventhal EL, Crisco JJ, Fleming BC (2010) Automatic determination of anatomical coordinate systems for three-dimensional bone models of the isolated human knee. Journal of biomechanics 43:1623-1626; https://doi.org/10.1016/J.JBIOMECH.2010.01.036
62. Modenese L, Renault JB (2021) Automatic generation of personalised skeletal models of the lower limb from three-dimensional bone geometries. Journal of Biomechanics 116:110186-110186; https://doi.org/10.1016/J.JBIOMECH.2020.110186
63. Moon SW, Park SH, Lee BH, Oh M, Chang M, Ahn JH, et al. (2015) The Effect of Hinge Position on Posterior Tibial Slope in Medial Open-Wedge High Tibial Osteotomy. Arthroscopy 31:1128-1133; https://doi.org/10.1016/J.ARTHRO.2015.01.009
64. Nedopil AJ, Hernandez AM, Boone JM, Howell SM, Hull ML (2023) Correcting for distal femoral asymmetry is necessary to determine postoperative alignment deviations from planned alignment of the femoral component. Knee 42:193-199; https://doi.org/10.1016/j.knee.2023.01.013
65. Ohmori T, Kabata T, Kajino Y, Inoue D, Ueno T, Taga T, et al. (2022) Importance of Three-Dimensional Evaluation of Surgical Transepicondylar Axis in Total Knee Arthroplasty. The Journal of Knee Surgery 35:032-038; https://doi.org/10.1055/s-0040-1712087
66. Okamoto S, Mizu-uchi H, Okazaki K, Hamai S, Tashiro Y, Nakahara H, et al. (2016) Two-dimensional planning can result in internal rotation of the femoral component in total knee arthroplasty. Knee Surgery, Sports Traumatology, Arthroscopy 24:229-235; https://doi.org/10.1007/s00167-014-3370-1
67. Pangaud C, Laumonerie P, Dagneaux L, LiArno S, Wellings P, Faizan A, et al. (2020) Measurement of the Posterior Tibial Slope Depends on Ethnicity, Sex, and Lower Limb Alignment: A Computed Tomography Analysis of 378 Healthy Participants. Orthopaedic journal of sports medicine 8; https://doi.org/10.1177/2325967119895258
68. Preston B, Harris S, Villet L, Mattathil C, Cobb J, Rivière C (2022) The medial condylar wall is a reliable landmark to kinematically align the femoral component in medial UKA: an in-silico study. Knee Surgery, Sports Traumatology, Arthroscopy 30:3220-3227; https://doi.org/10.1007/s00167-021-06683-9
69. Qin J, Chen D, Xu Z, Shi D, Dai J, Jiang Q (2018) Evaluation of the Effect of the Sulcus Angle and Lateral to Medial Facet Ratio of the Patellar Groove on Patella Tracking in Aging Subjects with Stable Knee Joint. BioMed Research International 2018; https://doi.org/10.1155/2018/4396139
70. Renault JB, Aüllo-Rasser G, Donnez M, Parratte S, Chabrand P (2018) Articular-surface-based automatic anatomical coordinate systems for the knee bones. Journal of Biomechanics 80:171-178; https://doi.org/10.1016/J.JBIOMECH.2018.08.028
71. Roth T, Carrillo F, Wieczorek M, Ceschi G, Esfandiari H, Sutter R, et al. (2021) Three-dimensional preoperative planning in the weight-bearing state: validation and clinical evaluation. Insights into Imaging 12:1-11; https://doi.org/10.1186/s13244-021-00994-8
72. Roth T, Sigrist B, Wieczorek M, Schilling N, Hodel S, Walker J, et al. (2023) An automated optimization pipeline for clinical-grade computer-assisted planning of high tibial osteotomies under consideration of weight-bearing. Computer Assisted Surgery 28:2211728; https://doi.org/10.1080/24699322.2023.2211728
73. Sasaki R, Niki Y, Kaneda K, Yamada Y, Nagura T, Nakamura M, et al. (2023) Three-dimensional joint surface orientation does not correlate with two-dimensional coronal joint line orientation in knee osteoarthritis: Three-dimensional analysis of upright computed tomography. The Knee 43:10-17; https://doi.org/10.1016/j.knee.2023.05.001
74. Sato A, Takagi H, Koya T, Espinoza Orías AA, Kanzaki K, Inoue N (2023) Clinical three-dimensional anatomy of the femur considering navigation-aided surgery of total knee arthroplasty in Japanese patients. The Knee 41:214-220; https://doi.org/10.1016/j.knee.2022.12.001

75. Siboni R, Vialla T, Joseph E, LiArno S, Faizan A, Martz P, et al. (2022) Coronal and sagittal alignment of the lower limb in Caucasians: Analysis of a 3D CT database. Orthopaedics & Traumatology: Surgery & Research 108:103251-103251; https://doi.org/10.1016/j.otsr.2022.103251
76. Subburaj K, Ravi B, Agarwal M (2010) Computer-aided methods for assessing lower limb deformities in orthopaedic surgery planning. Computerized medical imaging and graphics 34:277-288; https://doi.org/10.1016/J.COMPMEDIMAG.2009.11.003
77. Tanoğlu O, Subaşı İÖ, Gökgöz MB, Arıcan G (2021) Is Proximal Tibia Sufficient for Accurate Measurement of Tibial Slope Angles on Three-dimensional Tomography-based Anatomical Models? Current Medical Imaging 17:1419-1424; https://doi.org/10.2174/1573405617666210806150938
78. Tarassoli P, Warnock JM, Lim YP, Jagota I, Parker D (2024) Large multiplanar changes to native alignment have no apparent impact on clinical outcomes following total knee arthroplasty. Knee Surgery, Sports Traumatology, Arthroscopy 32:432-444; https://doi.org/10.1002/ksa.12044
79. Teng Y, Mizu-uchi H, Xia Y, Akasaki Y, Akiyama T, Kawahara S, et al. (2021) Axial But Not Sagittal Hinge Axis Affects Posterior Tibial Slope in Medial Open-Wedge High Tibial Osteotomy: A 3-Dimensional Surgical Simulation Study. Arthroscopy 37:2191-2201; https://doi.org/10.1016/J.ARTHRO.2021.01.063
80. Tiefenboeck S, Sesselmann S, Taylor D, Forst R, Seehaus F (2022) Preoperative planning of total knee arthroplasty: reliability of axial alignment using a three-dimensional planning approach. Acta Radiologica 63:1051-1061; https://doi.org/10.1177/02841851211029076
81. Twiggs JG, Dickison DM, Kolos EC, Wilcox CE, Roe JP, Fritsch BA, et al. (2018) Patient Variation Limits Use of Fixed References for Femoral Rotation Component Alignment in Total Knee Arthroplasty. The Journal of Arthroplasty 33:67-74; https://doi.org/10.1016/j.arth.2017.08.023
82. Van Genechten W, Van Haver A, Bartholomeeusen S, Claes T, Van Beek N, Michielsen J, et al. (2023) Impacted bone allograft personalised by a novel 3D printed customization kit produces high surgical accuracy in medial opening wedge high tibial osteotomy: a pilot study. Journal of Experimental Orthopaedics 10:24; https://doi.org/10.1186/s40634-023-00593-0
83. Vanhove F, Noppe N, Fragomen AT, Hoekstra H, Vanderschueren G, Metsemakers WJ (2019) Standardization of torsional CT measurements of the lower limbs with threshold values for corrective osteotomy. Archives of Orthopaedic and Trauma Surgery 139:795-805; https://doi.org/10.1007/S00402-019-03139-1
84. Victor J, Van Doninck D, Labey L, Innocenti B, Parizel PM, Bellemans J (2009) How precise can bony landmarks be determined on a CT scan of the knee? The Knee 16:358-365; https://doi.org/10.1016/J.KNEE.2009.01.001
85. Vuurberg G, Dahmen J, Dobbe IGG, Kleipool RP, Hayat B, Sierevelt IN, et al. (2022) Lower leg symmetry: a Q3D-CT analysis. Surgical and Radiologic Anatomy 44:851-860; https://doi.org/10.1007/s00276-022-02940-9
86. Wai Hung CL, Wai Pan Y, Kwong Yuen C, Hon Bong L, Lei Sha LW, Ho Man SW (2009) Interobserver and intraobserver error in distal femur transepicondylar axis measurement with computed tomography. The Journal of arthroplasty 24:96-100; https://doi.org/10.1016/J.ARTH.2007.11.014
87. Wakelin EA, Tran L, Twiggs JG, Theodore W, Roe JP, Solomon MI, et al. (2018) Accurate determination of post-operative 3D component positioning in total knee arthroplasty: the AURORA protocol. Journal of Orthopaedic Surgery and Research 13:275; https://doi.org/10.1186/s13018-018-0957-0
88. Xing Q, Han R, Li Y, Yang W, Chen JX (2013) Automatically assessing limb alignment and hip fracture using 3D models. Computing in Science and Engineering 15:10-20; https://doi.org/10.1109/MCSE.2012.107
89. Yamagami R, Inui H, Taketomi S, Kono K, Kawaguchi K, Sameshima S, et al. (2022) Proximal tibial morphology is associated with risk of trauma to the posteromedial structures during tibial bone resection reproducing the anatomical posterior tibial slope in bicruciate-retaining total knee arthroplasty. The Knee 36:1-8; https://doi.org/10.1016/j.knee.2022.03.008
90. Yang G, Wang Z, Wen X, Jiang Z, Qi X, Yang C (2016) The relationship between the midpoints connecting the tibial attachments of the anterior and posterior cruciate ligaments and the transepicondylar axis: In vivo three-dimensional measurement in the Chinese population. The Knee 23:777-784; https://doi.org/10.1016/J.KNEE.2016.05.003
91. Yang Y, Zeng X, Jin Y, Zhu Z, Tsai T-Y, Chen J, et al. (2022) The Presence of Cartilage Affects Femoral Rotational Alignment in Total Knee Arthroplasty. Frontiers in Surgery 9; https://doi.org/10.3389/fsurg.2022.802631
92. Yue B, Varadarajan KM, Ai S, Tang T, Rubash HE, Li G (2011) Differences of knee anthropometry between Chinese and white men and women. The Journal of arthroplasty 26:124-130; https://doi.org/10.1016/J.ARTH.2009.11.020
93. Zhang LS, Zhou H, Zhang JC, Zhang Q, Chen XY, Feng S (2022) Different tibial rotational axes can be applied in combination according to the tibial tuberosity-posterior cruciate ligament distance in total knee arthroplasty. BMC Musculoskeletal Disorders 23:906; https://doi.org/10.1186/s12891-022-05859-9

94. Zhang R-Y, Su X-Y, Zhao J-X, Li J-T, Zhang L-C, Tang P-F (2020) Three-dimensional morphological analysis of the femoral neck torsion angle-an anatomical study. Journal of orthopaedic surgery and research 15:192-192; https://doi.org/10.1186/s13018-020-01712-8
95. Zhang Y, Wang J, Xiao J, Zhao L, Li Zh, Yan G, et al. (2014) Measurement and comparison of tibial posterior slope angle in different methods based on three-dimensional reconstruction. The Knee 21:694-698; https://doi.org/10.1016/J.KNEE.2014.01.008
